# CD4^+^ T helper 2 cells suppress breast cancer by inducing terminal differentiation

**DOI:** 10.1084/jem.20201963

**Published:** 2022-06-03

**Authors:** Margherita Boieri, Anna Malishkevich, Ranya Guennoun, Emanuela Marchese, Sanne Kroon, Kathryn E. Trerice, Mary Awad, Jong Ho Park, Sowmya Iyer, Johannes Kreuzer, Wilhelm Haas, Miguel N. Rivera, Shadmehr Demehri

**Affiliations:** 1 Center for Cancer Immunology and Cutaneous Biology Research Center, Department of Dermatology, Massachusetts General Hospital and Harvard Medical School, Boston, MA; 2 Center for Cancer Research, Massachusetts General Hospital and Harvard Medical School, Boston, MA; 3 Department of Pathology, Massachusetts General Hospital and Harvard Medical School, Boston, MA; 4 Department of Medicine, Massachusetts General Hospital and Harvard Medical School, Boston, MA

## Abstract

Cancer immunology research is largely focused on the role of cytotoxic immune responses against advanced cancers. Herein, we demonstrate that CD4^+^ T helper (Th2) cells directly block spontaneous breast carcinogenesis by inducing the terminal differentiation of the cancer cells. Th2 cell immunity, stimulated by thymic stromal lymphopoietin, caused the epigenetic reprogramming of the tumor cells, activating mammary gland differentiation and suppressing epithelial–mesenchymal transition. Th2 polarization was required for this tumor antigen–specific immunity, which persisted in the absence of CD8^+^ T and B cells. Th2 cells directly blocked breast carcinogenesis by secreting IL-3, IL-5, and GM-CSF, which signaled to their common receptor expressed on breast tumor cells. Importantly, Th2 cell immunity permanently reverted high-grade breast tumors into low-grade, fibrocystic-like structures. Our findings reveal a critical role for CD4^+^ Th2 cells in immunity against breast cancer, which is mediated by terminal differentiation as a distinct effector mechanism for cancer immunoprevention and therapy.

## Teaser

Boieri et al. demonstrate that CD4^+^ T helper cells directly block breast cancer development by forcing the cancer cells to terminally differentiate.

## Graphical Abstract

**Figure:**
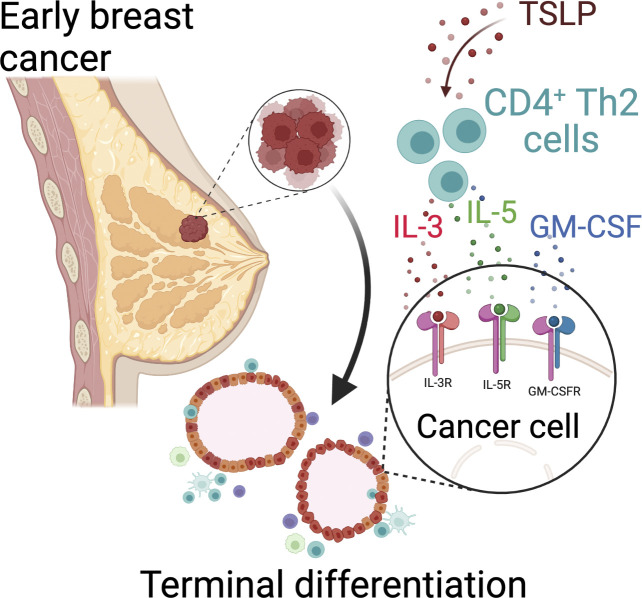

## Introduction

Advances in the field of cancer immunology have led to the advent of novel immunotherapeutics that reactivate tumor-infiltrating CD8^+^ cytotoxic T lymphocytes (CTLs) against late-stage metastatic cancers such as melanoma (Mellman et al., 2011). However, the potential efficacy of activating the immune system against early-stage cancers is largely unexplored. This is particularly relevant to breast and other epithelial cancers because these malignancies frequently lack any significant immune infiltrate at baseline, especially during the early in situ phases of their development (Finn, 2018). The current cancer immunotherapies, such as immune checkpoint blockade, which rely on a pre-existing T cell infiltrate in the tumor for their effects, have low efficacy against nonimmunogenic epithelial cancers with an immunosuppressive microenvironment (Finn, 2018). In addition, the antigen-directed immunotherapeutics, including neoantigen-based vaccines and engineered T cells, have limited applicability in an early cancer with a low mutational load (Yarchoan et al., 2017). These challenges highlight the urgent need for novel approaches to enable the use of the immune system for cancer immunoprevention.

Breast cancer is the most common cancer in women, with >1.7 million new diagnoses per year across the globe (Ferlay et al., 2015). The mainstay treatments for breast cancer are associated with significant side effects, and acquired resistance remains a major limitation to their use. In addition, the current treatment modalities have a limited ability to prevent cancer development in high-risk patients or block the long-term risk of breast cancer recurrence, which affects ≤30% of women with invasive breast cancer (Harris et al., 2000; Welch et al., 2000) and 19% of women with ductal carcinoma in situ (Solin et al., 2005). In contrast, activation of adaptive immunity, sufficient to block breast cancer formation from precancerous lesions, has the potential to provide long-term therapeutic efficacy for breast cancer prevention and a durable cure.

CD4^+^ T helper (Th) cells play a central role in coordinating the adaptive immune responses at epithelial sites by releasing a wide array of cytokines that recruit and regulate the activity of other immune cells (Kennedy and Celis, 2008; Zhu, 2018). Among Th cell subsets, Th1 cells orchestrate cell-mediated immunity against advanced cancers through the production of IFNγ, TNFα, and other inflammatory mediators to directly target cancer cells and facilitate the recruitment and activation of CTLs and natural killer (NK) cells against cancer (Braumüller et al., 2013; Kennedy and Celis, 2008). In contrast, Th2 cells have been linked to tumor promotion in the context of chronic inflammation by activating humoral immune responses and interfering with recruitment and activation of CTLs in cancer (Johansson et al., 2008). However, epidemiological studies have found that patients with allergic diseases, which are driven by inflammatory Th2 cells, are less susceptible to developing breast cancer (Bozek et al., 2020; Hedderson et al., 2003; Vena et al., 1985; Wang and Diepgen, 2005). Thus, defining the precise mechanism by which Th2 cell immunity can suppress early malignant transformation in mammary glands will have major implications for breast cancer prevention in high-risk individuals.

Previously, we have demonstrated that thymic stromal lymphopoietin (TSLP), an epithelium-derived cytokine and a master regulator of allergic inflammation in barrier organs (Divekar and Kita, 2015; Ziegler, 2012), blocks breast cancer development through the activation of CD4^+^ T cells (Demehri et al., 2016). Herein, we investigated the mechanism by which TSLP-stimulated CD4^+^ T cells suppress breast carcinogenesis. We found that Th2 polarization was required to protect the breast from oncogene-driven malignant progression, even in the absence of CD8^+^ T and B cells. Tumor antigen–specific Th2 cells directly induced an epigenetic reprogramming of the breast cancer cells, which blocked their epithelial–mesenchymal transition (EMT) and promoted the expression of the genes involved in normal mammary gland development. We show that IL-3, IL-5, and GM-CSF released by Th2 cells are responsible for the induction of terminal differentiation in the developing breast cancer and demonstrate that Th2 cell immunity reverted high-grade breast cancers into low-grade, fibrocystic-like structures. Finally, we show the loss of TSLP during human breast cancer development and demonstrate significantly improved survival in patients with high *TSLP* expression in their breast cancer. Collectively, our findings establish a previously unrecognized role for Th2 cells in immunity against breast carcinogenesis and highlight terminal differentiation as a novel immune effector mechanism for cancer prevention and therapy.

## Results

### TSLP-induced CD4^+^ T cell immunity transforms high-grade breast tumors into fibrocystic structures

**Figure 1.**
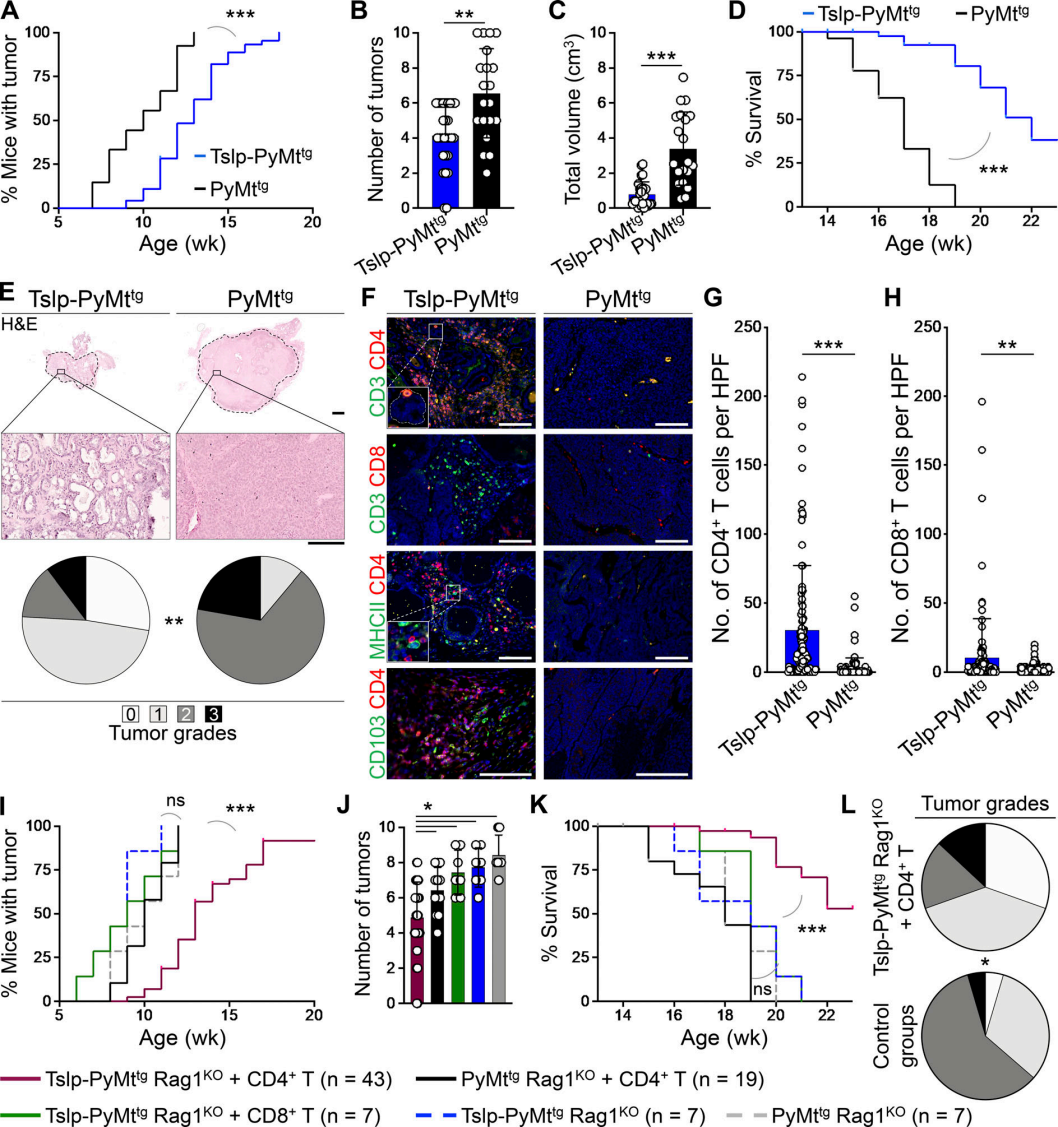
**TSLP-induced CD4**^**+**^ **T cell immunity transforms high-grade breast tumors into fibrocystic structures. (A)** Time to tumor onset in K*14-Tslp*^*tg*^*, MMTV-PyMT*^*tg*^ (Tslp-PyMt^tg^; *n* = 46) and PyMt^tg^ (*n* = 27) mice (log-rank test). **(B)** Number of palpable tumors in each Tslp-PyMt^tg^ (*n* = 28) and PyMt^tg^ (*n* = 20) mouse at 18–20 wk of age (Mann–Whitney *U* test). Bar graph shows mean + SD. **(C)** Sum of the volumes of all tumors in each Tslp-PyMt^tg^ (*n* = 28) and PyMt^tg^ (*n* = 20) mouse at 18–20 wk of age (Mann–Whitney *U* test). Bar graph shows mean + SD. **(D)** Percentage survival of Tslp-PyMt^tg^ (*n* = 46) and PyMt^tg^ (*n* = 27) mice (log-rank test). **(E)** Representative images of H&E-stained primary Tslp-PyMt^tg^ and PyMt^tg^ tumors at low (scale bar: 1 mm) and high (scale bar: 100 μm) magnifications, and the distribution of histological grades of Tslp-PyMt^tg^ (*n* = 29) and PyMt^tg^ (*n* = 18) breast tumors (Fisher’s exact test). **(F)** Representative images of CD3/CD4, CD3/CD8, MHCII/CD4, and CD103/CD4 immunofluorescence staining on Tslp-PyMt^tg^ and PyMt^tg^ tumors (scale bar: 100 μm). Inset in CD3/CD4 image shows a CD4^+^ T cell within the epithelial compartment of a differentiated Tslp-PyMt^tg^ breast tumor. Inset in MHCII/CD4 image highlights the interaction of CD4^+^ T cells with the APCs within Tslp-PyMt^tg^ tumor microenvironment. **(G and H)** Quantification of CD4^+^ T cells (G) and CD8^+^ T cells (H) in Tslp-PyMt^tg^ and PyMt^tg^ tumors. CD3/CD4 and CD3/CD8 double-positive cells counted in 10 HPF images in 11 tumor samples per group. HPF images for each sample were chosen randomly across the tumor section. Each dot represents one HPF image (unpaired *t* test). Bar graphs show mean + SD. **(I)** Time to tumor onset in Tslp-PyMt^tg^ Rag1^KO^ + CD4^+^ T cells, PyMt^tg^ Rag1^KO^ + CD4^+^ T cells, Tslp-PyMt^tg^ Rag1^KO^ + CD8^+^ T cells, Tslp-PyMt^tg^ Rag1^KO^, and PyMt^tg^ Rag1^KO^ T cells (P < 0.0001 for Tslp-PyMt^tg^ Rag1^KO +^CD4^+^ compared with PyMt^tg^ Rag1^KO +^CD4^+^ T cell; no statistical difference among the other groups [ns], log-rank test). **(J)** Number of palpable tumors at endpoint (≥18 wk old) in the five groups of mice (Mann–Whitney *U* test). Bar graph shows mean + SD. **(K)** Percentage survival in the five groups of animals (P < 0.0001 for Tslp-PyMt^tg^ Rag1^KO^ + CD4^+^ compared with PyMt^tg^ Rag1^KO^ + CD4^+^ T cell, no statistical difference among the other groups [ns]; log-rank test). **(L)** Distribution of histological grades of Tslp-PyMt^tg^ Rag1^KO^ + CD4^+^ T cell tumors compared with all other groups combined (control groups, Fisher’s exact test). Each of the tumors in the studies is from a separate mouse. All experimental data verified in at least two independent experiments. *, P < 0.05; **, P < 0.01; ***, P < 0.0001.

**Figure S1.**
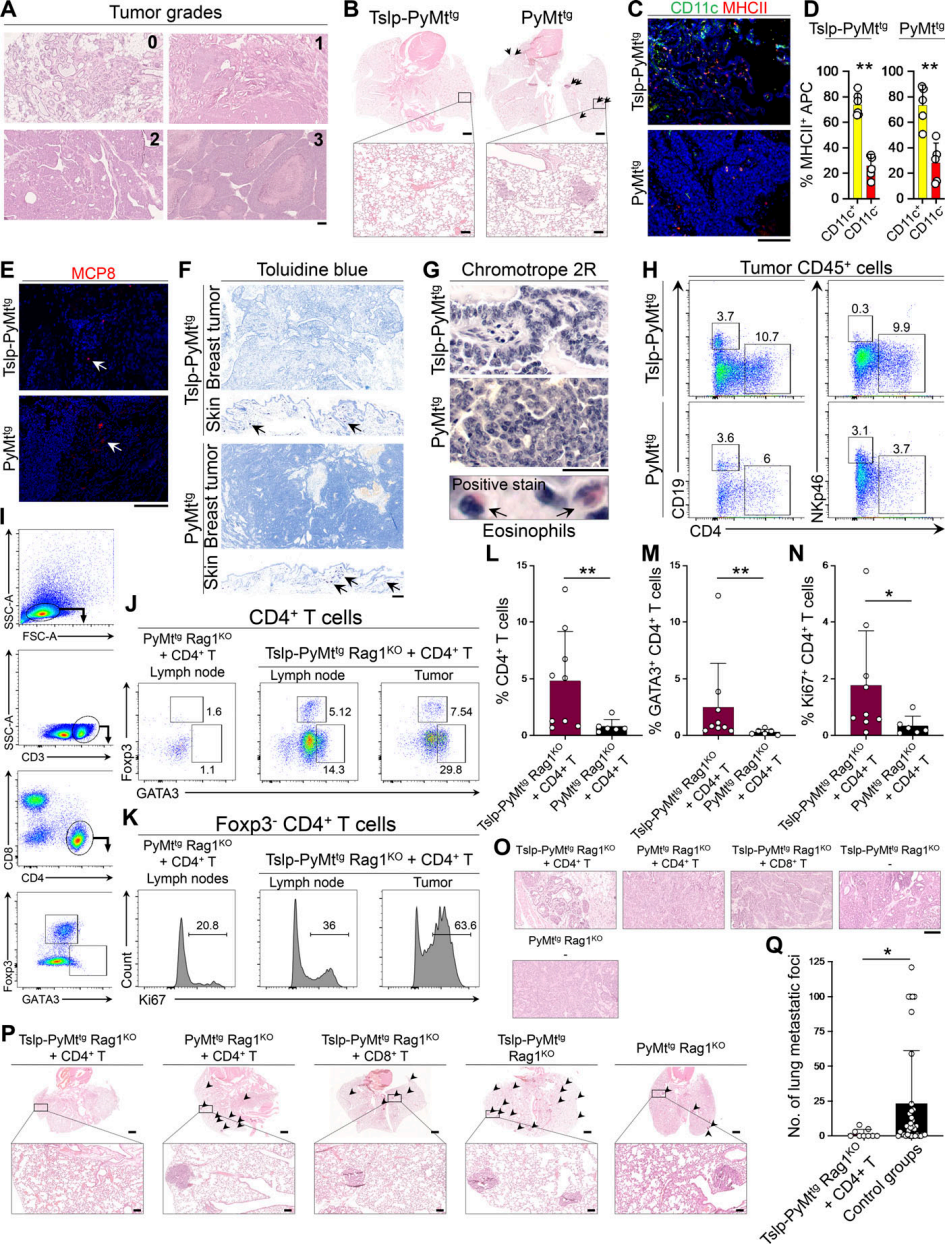
**TSLP induces CD4**^**+**^ **T cell immunity against spontaneous breast carcinogenesis. (A)** Representative images of H&E-stained mouse spontaneous breast tumors depicting tumor grades used in the study (scale bar: 100 μm). **(B)** Representative low (scale bars: 1 mm) and high (insets, scale bars: 100 μm) magnification images of H&E-stained lungs of Tslp-PyMt^tg^ and PyMt^tg^ mice. Arrows point to breast cancer metastatic foci in PyMt^tg^ lung. **(C)** Representative images of CD11c/MHCII immunofluorescence staining in Tslp-PyMt^tg^ and PyMt^tg^ breast tumors (scale bar: 100 μm). **(D)** Percentage CD11c^+^ MHCII^+^ versus CD11c^−^ MHCII^+^ APCs in Tslp-PyMT^tg^ (*n* = 6) and PyMT^tg^ (*n* = 5) breast tumors (Mann–Whitney *U* test). **(E)** Representative images of MCP8 immunofluorescence staining for basophils in Tslp-PyMt^tg^ and PyMt^tg^ tumors. Arrows highlight rare basophils in tumors (scale bar: 100 μm). **(F)** Representative images of toluidine blue staining for mast cells in Tslp-PyMt^tg^ and PyMt^tg^ breast tumors. Skin tissues from the same mice are included to show examples of positive staining for mast cells (arrows; scale bar: 100 μm). **(G)** Representative images of chromotrope 2R staining for eosinophil detection in Tslp-PyMt^tg^ and PyMt^tg^ breast tumors (scale bar: 100 μm). Positive stained eosinophils (arrows) in Tslp-PyMt^tg^ skin are highlighted with arrows in the lower panel. **(H)** Representative flow plots showing percentage CD19^+^ B cells and Nkp46^+^ NK cells among Tslp-PyMt^tg^ and PyMt^tg^ tumor-infiltrating CD45^+^ leukocytes. **(I)** Representative flow plots demonstrating the gating strategy used to assess GATA3^+^ CD4^+^ T cells in mice tissues. FSC, forward scatter; SSC, side scatter. **(J and K)** Representative flow cytometry dot plots (J) showing percentage GATA3 and Foxp3 positive cells (gated on CD3^+^CD4^+^ cells) in PyMt^tg^ Rag1^KO^ + CD4^+^ T cell lymph nodes versus Tslp-PyMt^tg^ Rag1^KO^ + CD4^+^ T cell lymph nodes and breast tumor and histograms (K) showing percentage Ki67^+^ cells among Foxp3^−^ CD4^+^ T effector cells in the two groups. Numbers on the plots highlight the percentage of cells within each gate. **(L–N)** Percentage CD4^+^ T (L), GATA3^+^ Th2 (M), and Ki67^+^ CD4^+^ (N) T cells in Tslp-PyMt^tg^ Rag1^KO +^CD4^+^ T cell (test, *n* = 9) versus PyMt^tg^ Rag1^KO^ + CD4^+^ T cell (control, *n* = 6) breast tumors. Note that the quantifications were performed in 10 HPF images per tumor sample stained with the respective markers. Each dot represents a tumor sample (Mann–Whitney *U* test). **(O)** Representative images of H&E-stained breast tumors from Tslp-PyMt^tg^ Rag1^KO^ + CD4^+^, PyMt^tg^ Rag1^KO^ + CD4^+^, Tslp-PyMt^tg^ Rag1^KO^ + CD8^+^, Tslp-PyMt^tg^ Rag1^KO^, and PyMt^tg^ Rag1^KO^ T cell groups (scale bar: 100 μm). **(P)** Representative low (scale bars: 1 mm) and high (inset, scale bars: 100 μm) magnification of H&E-stained lungs from Tslp-PyMt^tg^ Rag1^KO^ + CD4^+^ T cell, PyMt^tg^ Rag1^KO^ + CD4^+^ T cell, Tslp-PyMt^tg^ Rag1^KO^ + CD8^+^ T cell, Tslp-PyMt^tg^ Rag1^KO^, and PyMt^tg^ Rag1^KO^ mice. Arrowheads point to the breast cancer metastatic foci in the lungs. **(Q)** The number of breast cancer metastatic foci in the lungs of Tslp-PyMt^tg^ Rag1^KO^ + CD4^+^ T cell mice (test, *n* = 9) compared with other groups combined (control groups, *n* = 35, Mann–Whitney *U* test). Bar graphs show mean + SD. All experimental data verified in at least two independent experiments. *, P < 0.05; **, P < 0.01.

To determine the mechanism by which TSLP-stimulated CD4^+^ T cells suppressed breast carcinogenesis, we examined spontaneous breast cancer development in *K14-Tslp*^*tg*^, *MMTV-PyMT*^*tg*^ (Tslp-PyMt^tg^) mice compared with PyMt^tg^ controls on the BALB/c background. Tslp-PyMt^tg^ mice develop allergic inflammation in the skin starting from 10 wk of age (Demehri et al., 2016). Tslp-PyMt^tg^ mice had delayed tumor onset (P < 0.0001; Fig. 1 A), developed fewer tumors (P = 0.0016; Fig. 1 B), and had less tumor burden (P < 0.0001, Fig. 1 C) associated with markedly extended survival compared with PyMt^tg^ animals (P < 0.0001, Fig. 1 D). Analysis of PyMt^tg^ breast tumors harvested at 18–20 wk of age showed high histological grades including compact collection of tumor cells with necrotic foci and the absence of any structures resembling a mammary gland (Figs. 1 E and S1 A). In stark contrast, the majority of the tumors from Tslp-PyMt^tg^ mice of the same age showed low histological grades resembling benign fibrocystic breast changes instead of adenocarcinoma and lacked metastatic potential (P = 0.0005; Figs. 1 E and S1 B). Massive infiltration of CD4^+^ T cells including few CD103^+^ CD4^+^ tissue resident memory T (T_RM_) cells and APCs surrounded Tslp-PyMt^tg^ tumor foci, while very few CD4^+^ T cells were detectable in the PyMt^tg^ tumors (P < 0.0001; Fig. 1, F and G). A smaller CD8^+^ T cell infiltrate was also present in Tslp-PyMt^tg^ compared with PyMt^tg^ tumors (P = 0.0009; Fig. 1, F and H). Conventional CD11c^+^ dendritic cells constituted the majority of APCs in Tslp-PyMt^tg^ tumors (Fig. S1, C and D; Demehri et al., 2016). We detected low numbers of basophils, mast cells, eosinophils, B cells, and NK cells in Tslp-PyMt^tg^ and PyMt^tg^ tumors (Fig. S1, E–H). These findings demonstrate that TSLP-stimulated CD4^+^ T cell immunity is associated with a halt in breast cancer progression and the development of low-grade fibrocystic structures.

### TSLP-activated CD4^+^ T cells are sufficient to block breast carcinogenesis in the absence of CD8^+^ T and B cells

To investigate the role of adaptive immune cells in TSLP-induced breast cancer suppression, we transferred naive CD4^+^ or CD8^+^ T cells from WT mice into *MMTV-PyMT*^*tg*^*, Rag1*^*−/−*^ (PyMt^tg^ Rag1^KO^) animals with or without *K14-Tslp* transgene. In the absence of T and B cells, TSLP overexpression had no impact on breast cancer development in Tslp-PyMt^tg^ Rag1^KO^ compared with PyMt^tg^ Rag1^KO^ mice (Fig. 1, I–K). However, CD4^+^ T cell transfer into Tslp-PyMt^tg^ Rag1^KO^ reconstituted breast cancer protection in these animals, as shown by significantly delayed tumor onset (P < 0.0001; Fig. 1 I), fewer tumors (P < 0.05; Fig. 1 J), and markedly increased survival compared with PyMt^tg^ Rag1^KO^ mice that received CD4^+^ T cells (P < 0.0001; Fig. 1 K). The transferred CD4^+^ T cells became highly proliferating GATA3^+^ CD4^+^ T cells in the tumor-draining lymph nodes and breast tumors of Tslp-PyMt^tg^ Rag1^KO^ mice (Fig. S1, I–N). Unlike the CD4^+^ T cell transfer group, Tslp-PyMt^tg^ Rag1^KO^ mice that received CD8^+^ T cells did not gain any protection against breast cancer (Fig. 1, I–K). Breast tumors of Tslp-PyMt^tg^ Rag1^KO^ mice that received CD4^+^ T cells had significantly lower histological grades and lacked metastatic potential compared with control groups (P = 0.0149; Figs. 1 L and S1, O–Q). Although we cannot rule out a role for CD8^+^ T cells, these findings demonstrate that CD4^+^ T cells are sufficient to deliver the TSLP-induced antitumor immunity in the breast in the absence of CD8^+^ T and B cells.

### CD4^+^ T cell immunity causes the terminal differentiation of breast cancer cells

**Figure 2.**
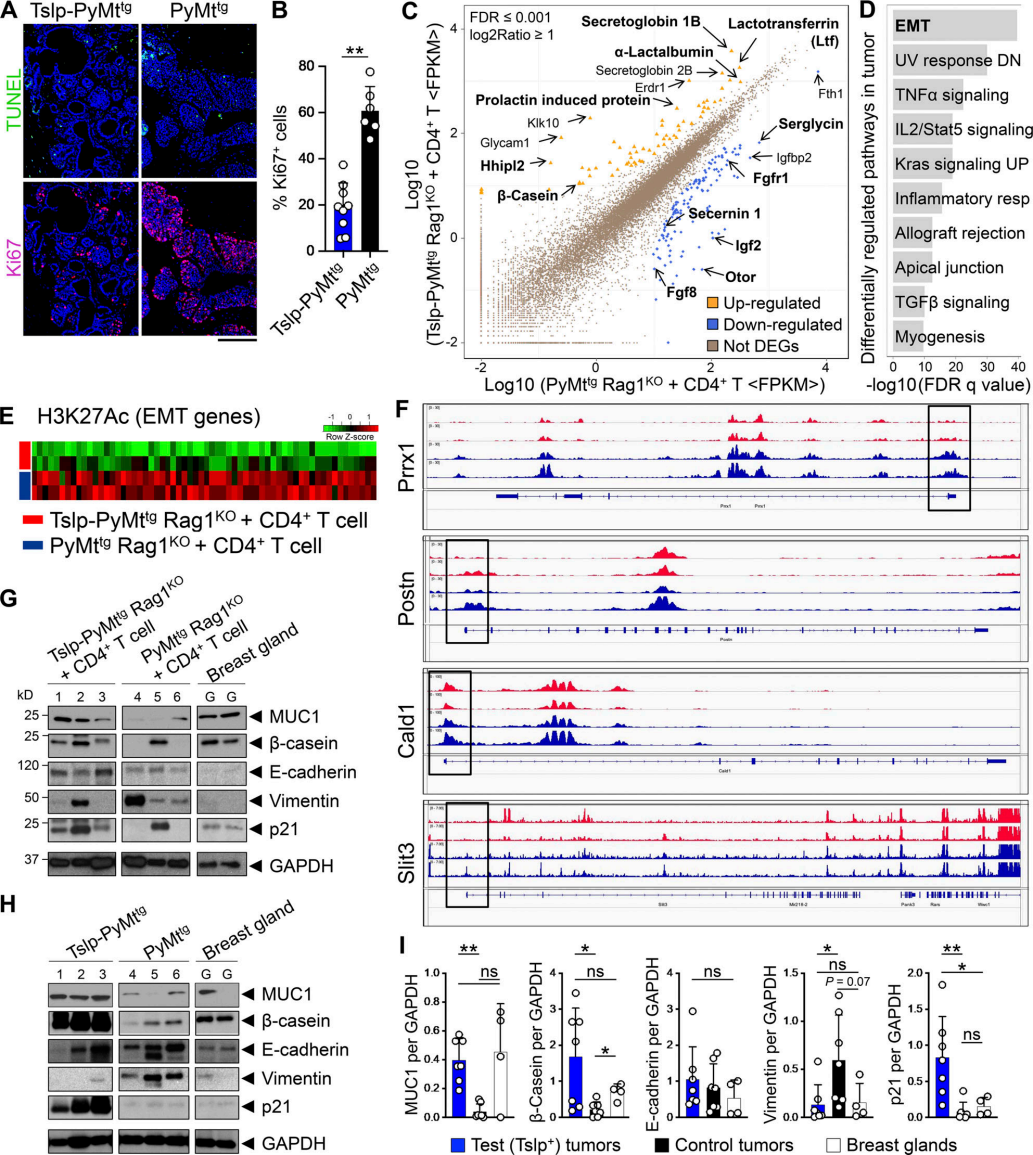
**CD4**^**+**^ **T cell immunity causes breast cancer terminal differentiation. (A)** Representative images of TUNEL assay (upper panels) and immunofluorescence staining for Ki67 (lower panels) on Tslp-PyMt^tg^ and PyMt^tg^ tumors (scale bar: 100 μm). **(B)** Percentage Ki67^+^ cells in Tslp-PyMT^tg^ (*n* = 9) and PyMT^tg^ (*n* = 6) breast tumors. Quantifications were performed in 10 HPF images per tumor sample stained with Ki67. Each dot represents a tumor sample (Mann–Whitney *U* test). Bar graph shows mean + SD. **(C)** Scatter plot showing genes differentially regulated in Tslp-PyMt^tg^ Rag1^KO^ + CD4^+^ T cell compared with PyMt^tg^ Rag1^KO^ + CD4^+^ T cell. Dots highlighted in yellow and blue represent genes up- or down-regulated in Tslp-PyMt^tg^ Rag1^KO^ + CD4^+^ T cell versus PyMt^tg^ Rag1^KO^ + CD4^+^ T cell, respectively (*n* = 3 per group, false discovery rate ≤ 0.001 and log_2_ ratio ≥ 1). Genes of interest are indicated with their symbol or complete name. **(D)** Pathway enrichment analysis of genes associated with differential H3K27Ac mark in the tumors of Tslp-PyMt^tg^ Rag1^KO^ + CD4^+^ T cell compared with PyMt^tg^ Rag1^KO^ + CD4^+^ T cells (*n* = 2 per group). **(E)** Heatmap showing increased H3K27Ac mark in the enhancer region of genes associated with EMT in PyMt^tg^ Rag1^KO^ + CD4^+^ T cell (control) compared with Tslp-PyMt^tg^ Rag1^KO^ + CD4^+^ T cell (test) group. **(F)** Peaks of acetylation in the promoter regions (highlighted by black boxes) of representative EMT genes in Tslp-PyMt^tg^ Rag1^KO^ + CD4^+^ T cells (red tracks) compared with PyMt^tg^ Rag1^KO^ + CD4^+^ T cells (blue tracks). **(G and H)** Western blot for breast epithelial (MUC1, β-casein, and E-cadherin), mesenchymal (vimentin), and regulator of proliferation (p21) markers on tumor lysates from Tslp-PyMt^tg^ Rag1^KO^ + CD4^+^ T cells, PyMt^tg^ Rag1^KO^ + CD4^+^ T cells, and normal mammary gland (G) and Tslp-PyMt^tg^, PyMt^tg^, and normal mammary gland (H). GAPDH is used as the control housekeeping protein. **(I)** Quantification of protein bands in Tslp-PyMt^tg^ Rag1^KO^ + CD4^+^ T cell and Tslp-PyMt^tg^ groups (test tumors) compared with PyMt^tg^ Rag1^KO^ + CD4^+^ T cell and PyMt^tg^ groups (control tumors) and WT breast glands (Mann–Whitney *U* test). Bar graphs show mean + SD. Each of the tumors in the studies is from a separate mouse. All experimental data verified in at least two independent experiments. *, P < 0.05; **, P < 0.01; ***, P < 0.0001.

**Figure S2.**
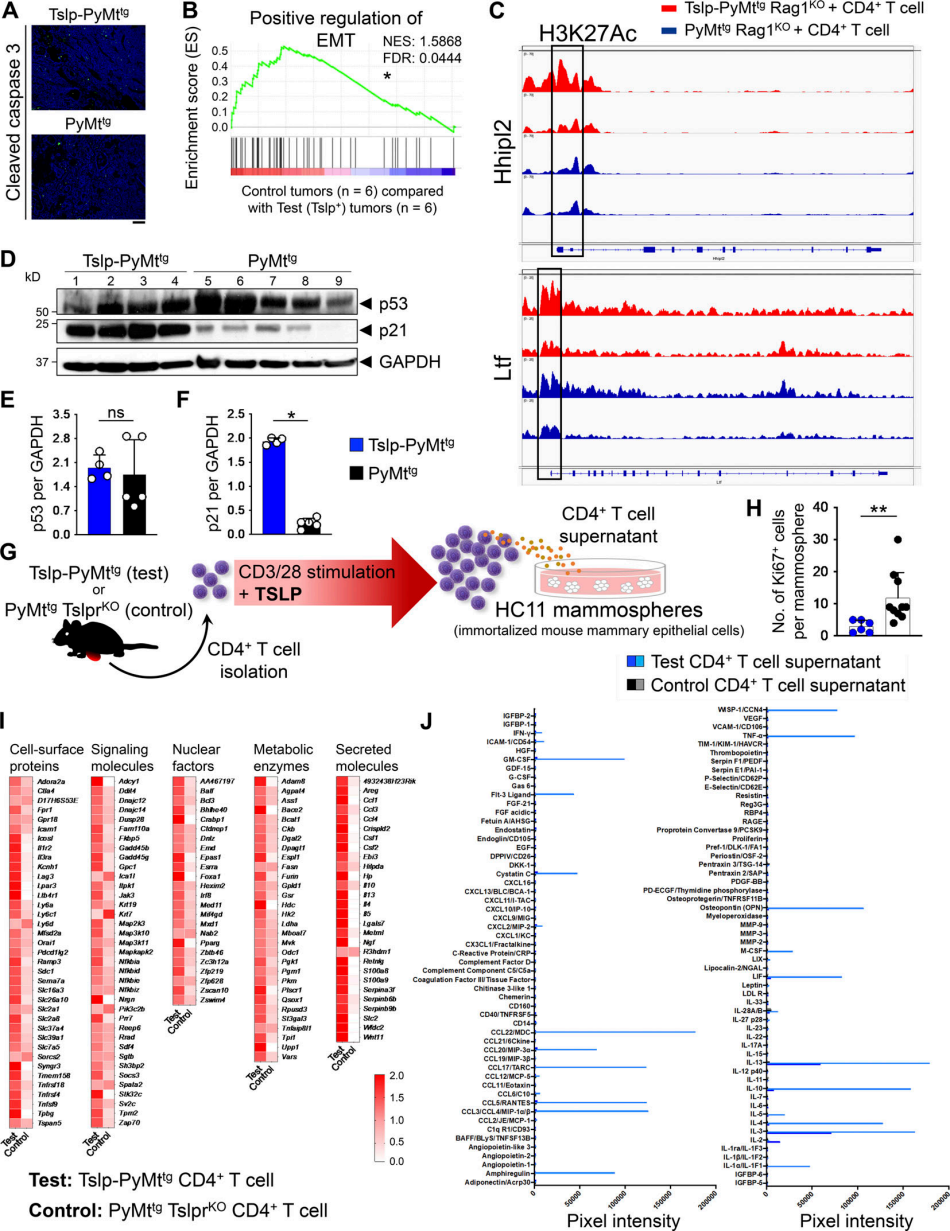
**CD4**^**+**^ **T cell immunity suppresses EMT and induces breast cancer differentiation. (A)** Representative immunofluorescence staining images of the apoptosis marker cleaved caspase 3 in Tslp-PyMt^tg^ and PyMt^tg^ breast tumors (scale bar: 100 μm). **(B)** The gene set enrichment analysis plot of positive regulation of EMT genes in PyMt^tg^ Rag1^KO^ + CD4^+^ T cell and PyMt^tg^ (control, *n* = 6) compared with *Tslp* overexpressing Tslp-PyMt^tg^ Rag1^KO^ + CD4^+^ T cell and Tslp-PyMt^tg^ breast tumors (test, *n* = 6) from RNA-sequencing data. NES, normalized enrichment score. **(C)** Peaks of H3K27 acetylation in the promoter region, highlighted by black boxes, of mammary gland differentiation genes that were upregulated in Tslp-PyMt^tg^ Rag1^KO^ + CD4^+^ T cell (test, red tracks) compared with PyMt^tg^ Rag1^KO^ + CD4^+^ T cell (control, blue tracks) breast tumors. **(D)** Western blot for p53 and p21 on tumor lysates from Tslp-PyMt^tg^ and PyMt^tg^ primary tumors. GAPDH is used as the control housekeeping protein. **(E and F)** Quantification of p53 (E) and p21 (F) protein bands in Tslp-PyMt^tg^ (test, *n* = 4) compared with PyMt^tg^ (control, *n* = 5) tumors (Mann–Whitney *U* test). **(G)** Schematic diagram of the experimental procedure for CD4^+^ T cell isolation, ex vivo stimulation, and HC11 mammosphere culture. **(H)** The number of Ki67^+^ cells per HC11 mammosphere exposed to supernatants derived from Tslp-PyMt^tg^ CD4^+^ T cells (test CD4^+^ T cell sup., *n* = 6) versus PyMt^tg^ Tslpr^KO^ CD4^+^ T cells (control CD4^+^ T cell sup., *n* = 10, Mann–Whitney *U* test). **(I)** The transcriptome of CD4^+^ T cells isolated from Tslp-PyMt^tg^ mice (test, *n* = 3) compared with CD4^+^ T cells isolated from PyMt^tg^ Tslpr^KO^ (control, *n* = 3) mice. The heatmap demonstrates the ratio between mean fragments per kilobase of transcript per million mapped reads (FPKM) value of each group and the average of mean FPKM values of the two groups. **(J)** Measurements of signal intensity of the cytokines, chemokines, and growth factors represented on the protein arrays performed on test (Tslp-PyMt^tg^) and control (PyMt^tg^ Tslpr^KO^) CD4^+^ T cells supernatants related to arrays shown in Fig. 3 E. Bar graph shows mean + SD. All experimental data verified in at least two independent experiments. *, P < 0.05; **, P < 0.01.

To determine the mechanism of breast cancer suppression by CD4^+^ T cells, we assessed apoptosis in early breast tumors of Tslp-PyMt^tg^ versus PyMt^tg^ mice using terminal deoxynucleotidyl transferase dUTP nick-end labeling (TUNEL) and cleaved caspase 3 assays. We did not detect any differences in apoptosis between Tslp-PyMt^tg^ and PyMt^tg^ tumors (Figs. 2 A and S2 A). Instead, we found a significant reduction in Ki67^+^ cells in Tslp-PyMt^tg^ tumors compared with PyMt^tg^ tumors (Fig. 2, A and B). To further define the impact of CD4^+^ T cell immunity on breast cancer cells, we compared the transcriptome of advanced breast tumors from Tslp-PyMt^tg^ Rag1^KO^ + CD4^+^ T cell (test) and PyMt^tg^ Rag1^KO^ + CD4^+^ T cell (control) groups. Among the differentially expressed genes, we found that β-casein (*Csn2*), lactotransferrin (*Ltf*), α-lactalbumin (*Lalba*), and other genes associated with milk production and mammary gland differentiation were upregulated in the test tumors, while genes associated with malignant progression including fibroblast growth factor receptor 1 (*Fgfr1*), insulin-like growth factor 2 (*Igf2*), and serglycin (*Srgn*) were upregulated in control tumors (Fig. 2 C). Genome-wide analysis of the acetylation at the 27th lysine residue of the histone H3 protein (H3K27Ac) using chromatin immunoprecipitation sequencing (ChIP-seq) on the same tumor samples used for RNA-sequencing revealed EMT to be the most differentially regulated pathway between the two groups (Fig. 2 D). Genes that are known to be upregulated during EMT had increased H3K27Ac in their enhancer and promoter regions in the control compared with test tumors (Fig. 2, E and F). Gene set enrichment analysis on the RNA sequencing data further confirmed the positive regulation of EMT gene set expression in control compared with test tumors (Fig. S2 B). In contrast, increased H3K27Ac was found on the promoter regions of genes associated with mammary gland development in test compared with control tumors (Fig. S2 C). To further examine the breast cancer differentiation versus EMT, we performed Western blot analysis on the protein lysates from test and control tumors as well as tumors from Tslp-PyMt^tg^ and PyMt^tg^ mice. Tslp-PyMt^tg^ Rag1^KO^ + CD4^+^ T cell and Tslp-PyMt^tg^ test tumors expressed higher levels of proteins associated with epithelial cell differentiation, Mucin 1 (MUC1), and mammary gland function, β-casein, in comparison to PyMt^tg^ Rag1^KO^ + CD4^+^ T cell and PyMt^tg^ control tumors (Fig. 2, G–I). Instead, control tumors expressed higher levels of the mesenchymal marker, vimentin, compared with Tslp-PyMt^tg^ Rag1^KO^ + CD4^+^ T cell and Tslp-PyMt^tg^ test tumors (Fig. 2, G–I). Consistent with the lack of proliferation associated with terminal differentiation, Tslp-PyMt^tg^ Rag1^KO^ + CD4^+^ T cell and Tslp-PyMt^tg^ test tumors expressed higher levels of p21 protein compared with PyMt^tg^ Rag1^KO^ + CD4^+^ T cell and PyMt^tg^ control tumors, which was independent of p53 overexpression (Fig. 2, G–I; and Fig. S2, D–F). These results demonstrate that CD4^+^ T cell immunity against early breast carcinogenesis is mediated by terminal differentiation and not by cellular cytotoxicity.

### CD4^+^ T cells directly block breast cancer development

**Figure 3.**
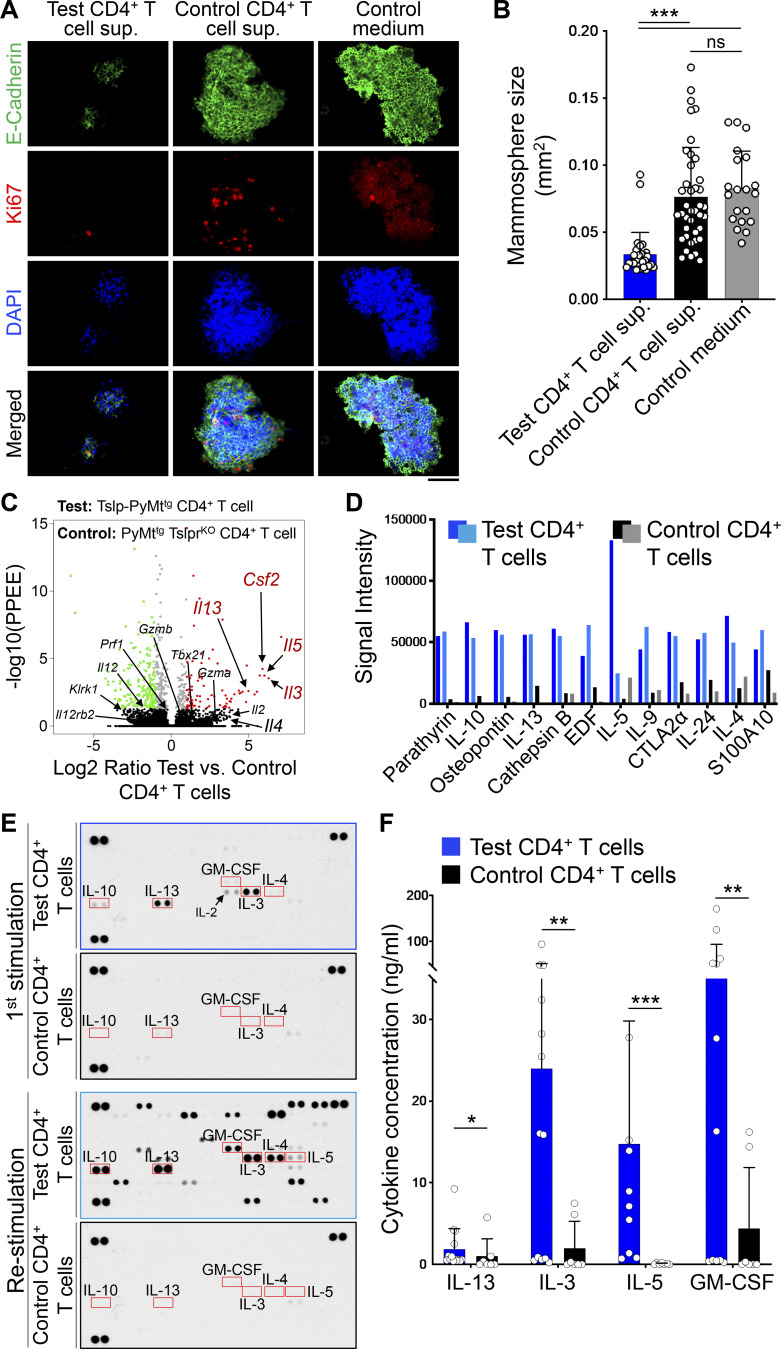
**CD4**^**+**^ **T cells directly suppress breast epithelial cell proliferation. (A)** Representative images of HC11 mammosphere immunofluorescence staining after exposure to supernatants derived from Tslp-PyMt^tg^ CD4^+^ T cells (test CD4^+^ T cell sup.), PyMt^tg^ Tslpr^KO^ CD4^+^ T cells (control CD4^+^ T cell sup.), or cell culture medium (control medium). Equal total protein amounts of supernatants from second and third rounds of CD4^+^ T cell stimulation were used. Mammospheres were stained for E-cadherin (green), Ki67 (red), and DAPI (blue, scale bar: 100 μm). **(B)** Quantification of mammosphere size in test CD4^+^ T cell sup. (*n* = 20), control CD4^+^ T cell sup. (*n* = 40), and control medium (*n* = 30) conditions. Each dot represents one mammosphere (Mann–Whitney *U* test). Bar graph shows mean + SD. **(C)** Volcano plot showing significantly upregulated (red dots) and downregulated (green dots) genes in CD4^+^ T cells isolated from Tslp-PyMt^tg^ mice (test, *n* = 6) compared with CD4^+^ T cells isolated from PyMt^tg^ Tslpr^KO^ mice (control, *n* = 4, log_2_ ratio > |1| and −log_10_ of posterior probability of equal expression [PPEE] >1.3 is considered significant). Genes of interest are indicated with their symbol. **(D)** Bar graph showing the top 12 secreted proteins detected in two Tslp-PyMt^tg^ CD4^+^ T cell (test) compared with two PyMt^tg^ Tslpr^KO^ CD4^+^ T cell (control) supernatants determined using multiplexed quantitative mass spectrometry. Supernatants were from second and third rounds of CD4^+^ T cell stimulation. **(E)** Image of protein arrays performed on CD4^+^ T cell supernatants from test (first and third arrays) and control (second and last arrays). Supernatants used in this assay were collected after a first round of CD4^+^ T cell stimulation ex vivo (top two arrays) or after the second round of stimulation (bottom two arrays). Equal total protein amount of each supernatant was used in this assay. Red boxes highlight Th2-related cytokines differentially detected in test versus control samples. **(F)** Quantification of protein concentration, measured with ELISA, in test CD4^+^ T cell supernatants compared with control CD4^+^ T cell supernatants. Supernatants used in this assay were collected after T cell stimulation. Supernatants were from first and second rounds of CD4^+^ T cell stimulation (Mann–Whitney *U* test). Bar graph shows mean + SD. The same amount of total protein for each sample was used for protein detection and quantification in D, E, and F. All experimental data verified in at least two independent experiments. *, P < 0.05; **, P < 0.01; ***, P < 0.0001.

To determine whether TSLP-stimulated CD4^+^ T cells can directly affect the differentiation of mammary epithelial cells, we developed a mammosphere/T cell culture system (Fig. S2 G). CD4^+^ T cells were sorted from tumors and tumor-draining lymph nodes of Tslp-PyMt^tg^ (test) or *MMTV-PyMT*^*tg*^*, Tslpr(Crlf2)*^*−/−*^ (PyMt^tg^ Tslpr^KO^; control) mice and stimulated ex vivo using anti-CD3/CD28 antibodies plus TSLP over one to three cycles. Supernatants were collected at the end of each stimulation cycle. The addition of TSLP to the culture is important to further stimulate CD4^+^ T cells ex vivo and simulate the conditions in Tslp-PyMt^tg^ mice. Because of the presence of TSLP in the culture, we used sorted CD4^+^ T cells from PyMt^tg^ Tslpr^KO^ mice as controls. A mouse mammary epithelial cell line, HC11, was exposed to supernatant from the T cells in a 3D mammosphere culture system and stained for E-cadherin, as a marker of epithelial cells, and Ki67 to mark proliferating cells. HC11 cells cultured in the supernatant from Tslp-PyMt^tg^ (test) CD4^+^ T cells formed small spherical mammospheres with low proliferation, while HC11 cells cultured in PyMt^tg^ Tslpr^KO^ (control) CD4^+^ T cell supernatant formed large and irregularly shaped mammospheres with increased cell proliferation, as marked by Ki67 expression (Fig. 3, A and B; and Fig. S2 H). These results indicate that CD4^+^ T cells from Tslp-PyMt^tg^ mice produce soluble factors responsible for blocking the proliferation while promoting the proper differentiation of mammary epithelial cells.

To examine the factors expressed by TSLP-stimulated CD4^+^ T cells, we performed RNA-sequencing on freshly sorted CD4^+^ T cells from Tslp-PyMt^tg^ (test) or PyMt^tg^ Tslpr^KO^ (control) mice. Th2 cell–associated gene sets were upregulated in the test compared with control CD4^+^ T cells (Fig. S2 I; Liu et al., 2020). Accordingly, test T cells had higher expression of genes encoding for Th2-related cytokines, IL-13 and IL-5 (Fig. 3 C). In addition, *Il3*, *Il5*, and *Csf2* scored among the top five upregulated genes in test versus control CD4^+^ T cells (Fig. 3 C). We did not detect any upregulation in genes associated with Th1 or cytotoxic T cell response (Fig. 3 C). To identify upregulated proteins secreted by test versus control T cells, we performed CD4^+^ T cell secretome analysis by labeling newly translated proteins with an azidohomoalanine tag that allowed their subsequent isolation and quantification using multiplexed quantitative mass spectrometry–based proteomics (Eichelbaum et al., 2012; McAlister et al., 2014). This analysis confirmed the presence of the Th2 cytokines, IL-13, IL-5, and IL-4, among the top 12 most abundantly secreted proteins in the test versus control CD4^+^ T cell supernatants and revealed the secretion of other Th2-associated cytokines such as IL-9, IL-10, and IL-24 by TSLP-activated CD4^+^ T cells (Fig. 3 D). To validate these results and capture other cytokines and chemokines that increased in test T cell supernatant, we tested CD4^+^ T cells supernatants on an array of 111 cytokines and chemokines. This assay clearly showed the induction of IL-3 and IL-13 in test T cell supernatant after first stimulation and additional increases in IL-4, IL-5, and GM-CSF levels after T cell restimulation (Figs. 3 E and S2 J). Finally, we quantified the elevated levels of IL-13, IL-3, IL-5, and GM-CSF in the supernatant derived from test compared with control CD4^+^ T cells (Fig. 3 F). These findings demonstrate that Th2-associated cytokines plus IL-3 and GM-CSF are prominent factors secreted by TSLP-stimulated CD4^+^ T cells.

### Th2 polarization is required for CD4^+^ T cell–mediated antitumor immunity in the breast

**Figure 4.**
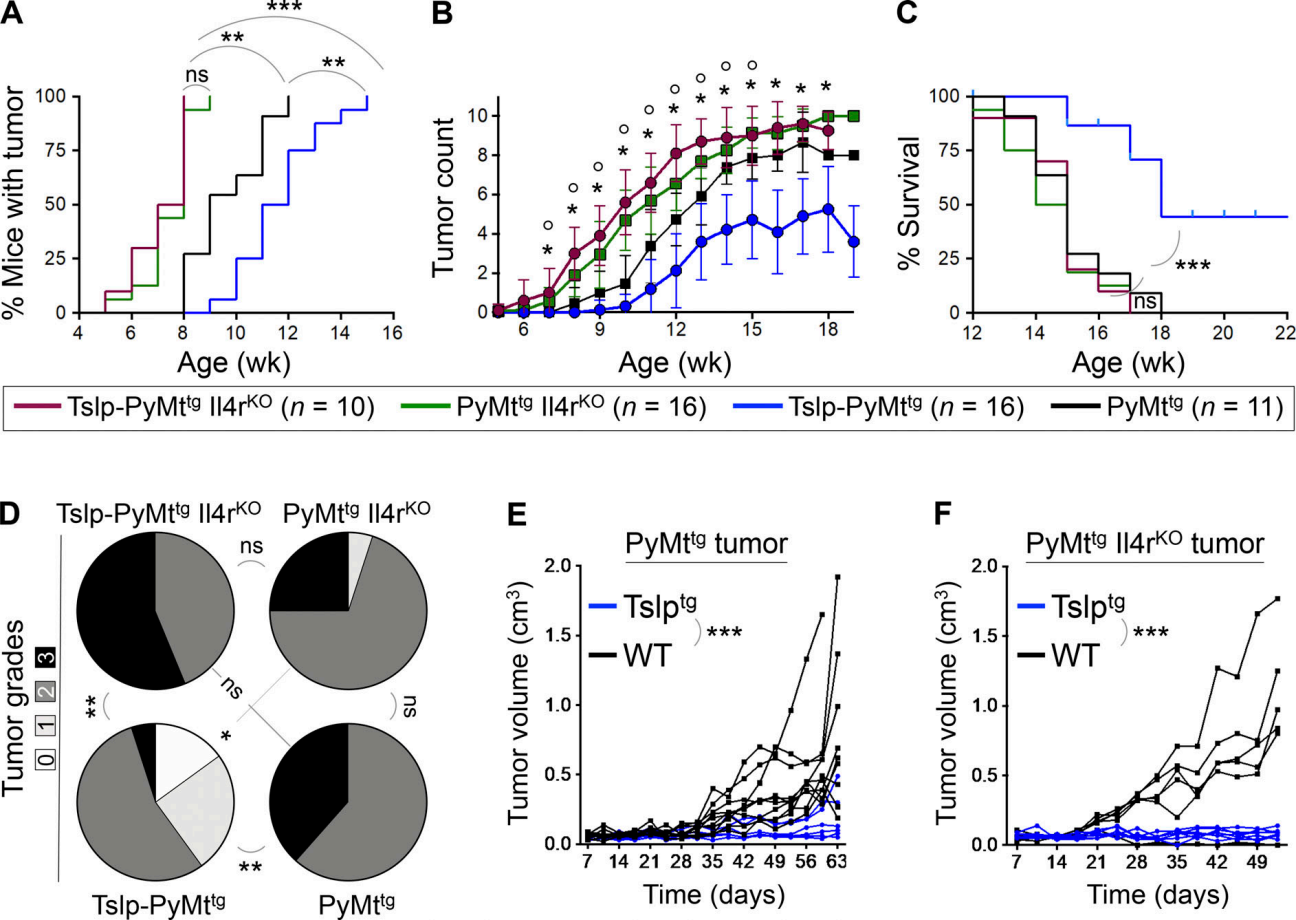
**Th2 cell polarization mediates the immunity against breast carcinogenesis. (A–C)** Comparison of tumor outcomes across Tslp-PyMt^tg^ Il4r^KO^, PyMt^tg^ Il4r^KO^, Tslp-PyMt^tg^, and PyMt^tg^ mice including time to tumor onset (log-rank test; A), number of tumors per mouse over time (*, P < 0.005 comparing Tslp-PyMt^tg^ Il4r^KO^ to Tslp-PyMt^tg^ and °, P < 0.05 comparing PyMt^tg^ Il4r^KO^ to PyMt^tg^, Mann–Whitney *U* test; B), and percentage survival of the animals in the four groups (log-rank test; C). **(D)** Distribution of histological grades of Tslp-PyMt^tg^ Il4r^KO^ (*n* = 16), PyMt^tg^ Il4r^KO^ (*n* = 20), Tslp-PyMt^tg^ (*n* = 20), and PyMt^tg^ (*n* = 13) tumors (Fisher’s exact test). **(E)** Tumor growth kinetic in Tslp^tg^ (test, *n* = 6, 6/6 tumors were <0.5 cm^3^ at the endpoint) versus WT (control, *n* = 10, 3/10 tumors were <0.5 cm^3^ at the endpoint) mice implanted with PyMt^tg^ primary breast tumors (two-way ANOVA). **(F)** Tumor growth kinetics in Tslp^tg^ (test, *n* = 7, 7/7 tumors were <0.5 cm^3^ at the endpoint) and WT (control, *n* = 5, 0/5 tumors were <0.5 cm^3^ at the endpoint) mice implanted with PyMt^tg^ Il4r^KO^ primary breast tumors (two-way ANOVA). Each of the tumors in the studies is from a separate mouse. All experimental data verified in at least two independent experiments. *, P < 0.05; **, P < 0.01; ***, P < 0.0001.

**Figure S3.**
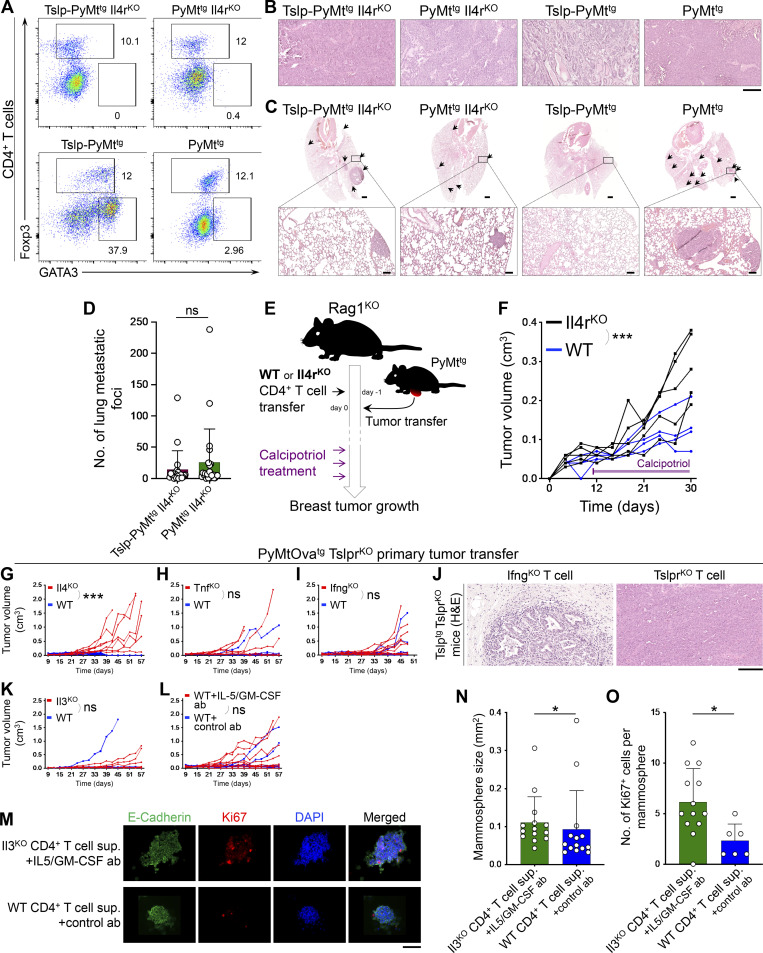
**Th2 polarization and cytokine release play critical roles in TSLP-stimulated CD4**^**+**^ **T cell immunity against breast cancer. (A)** Representative flow plots of transcriptional factor expression in tumor-infiltrating CD4^+^ T cells in Tslp-PyMt^tg^ Il4r^KO^, PyMt^tg^ Il4r^KO^, Tslp-PyMt^tg^, and PyMt^tg^ mice. Numbers on the plots show the percentage of the cells within each gate. **(B)** Representative images of H&E-stained breast tumors in Tslp-PyMt^tg^ Il4r^KO^, PyMt^tg^ Il4r^KO^, Tslp-PyMt^tg^, and PyMt^tg^ groups (scale bar: 100 μm). **(C)** Representative low (scale bars: 1 mm) and high (insets, scale bars: 100 μm) magnification images of H&E-stained lungs from Tslp-PyMt^tg^ Il4r^KO^, PyMt^tg^ Il4r^KO^, Tslp-PyMt^tg^, and PyMt^tg^ mice. Breast cancer metastatic foci in each lung are highlighted by arrows. **(D)** The number of breast cancer metastatic foci in the lungs of Tslp-PyMt^tg^ Il4r^KO^ (test, *n* = 20) compared with PyMt^tg^ Il4r^KO^ mice (control, *n* = 23, Mann–Whitney *U* test). **(E)** Schematic diagram of the experimental paradigm used to elucidate the ability of IL4rα^KO^ compared with WT CD4^+^ T cells to suppress breast tumor growth in Rag1^KO^ mice in response to TSLP induction by topical calcipotriol treatment. Note that the calcipotriol treatment was started when implanted PyMt^tg^ tumors became palpable (∼5 mm in diameter) and repeated every 3 d until the conclusion of the study. Tumor/T cell donor and recipient mice are on the BALB/c background. **(F)** Spider plot of PyMt^tg^ primary tumor growth in Rag1^KO^ mice injected with Il4rα^KO^ (*n* = 5) versus WT (*n* = 4) CD4^+^ T cells and treated with calcipotriol (two-way ANOVA). The duration of calcipotriol treatment is indicated by purple bar on the graph. **(G–I)** Spider plots of PyMtOva^tg^ Tslpr^KO^ primary tumor growth in Tslp^tg^ Tslpr^KO^ mice injected with Il4^KO^ (*n* = 8, 2/8 tumors were <0.5 cm^3^ at the endpoint; G), Tnf^KO^ (*n* = 6, 2/6 tumors were <0.5 cm^3^ at the endpoint; H), Ifng^KO^ (*n* = 10, 6/10 tumors were <0.5 cm^3^ at the endpoint; I) mutant CD4^+^ T cells compared to WT CD4^+^ T cells (*n* = 6, 6/6 tumors were <0.5 cm^3^; *n* = 6, 5/6 tumors were <0.5 cm^3^; and *n* = 8, 6/8 tumors were <0.5 cm^3^ at the endpoint, respectively). Note that Il4^KO^, Tnf^KO^, and Ifng^KO^ tumor growth data are also shown in Fig. 6, A–C (two-way ANOVA). **(J)** Representative images of H&E-stained PyMtOva^tg^ Tslpr^KO^ breast tumors in Tslp^tg^ Tslpr^KO^ mice injected with Ifng^KO^ versus Tslpr^KO^ CD4^+^ T cells (scale bar: 100 μm). **(K and L)** Spider plots of primary PyMtOva^tg^ Tslpr^KO^ tumor growth in Tslp^tg^ Tslpr^KO^ mice injected with Il3^KO^ (*n* = 6, 4/6 tumors were <0.5 cm^3^ at the endpoint) versus WT (*n* = 6, 5/6 tumors were <0.5 cm^3^ at the endpoint) CD4^+^ T cells (two-way ANOVA; K) and WT CD4^+^ T cells in combination with anti–IL-5 plus anti–GM-CSF blocking antibodies (ab, *n* = 9, 6/9 tumors were <0.5 cm^3^ at the endpoint) versus WT CD4^+^ T cells and Rat IgG isotype control antibody (*n* = 10, 8/10 tumors were <0.5 cm^3^ at the endpoint; L; two-way ANOVA). Note that Il3^KO^ and WT CD4^+^ T cells plus anti–IL-5 plus anti–GM-CSF blocking antibodies tumor growth data are also shown in Fig. 6, D and E. **(M)** Representative images of HC11 mammosphere immunofluorescence staining after exposure to supernatants (sup.) derived from splenic Il3^KO^ versus WT CD4^+^ T cells stimulated once with anti-CD3, anti-CD28, and TSLP for 3 d ex vivo. Before addition to HC11 mammosphere culture, Il3^KO^ and WT CD4^+^ T cell supernatants were mixed with IL5/GM-CSF blocking and control antibodies, respectively. Mammospheres were stained for E-cadherin (green), Ki67 (red), and DAPI (blue, scale bar: 100 μm). **(N)** Quantification of HC11 mammosphere size in Il3^KO^ CD4^+^ T cell supernatant plus IL-5/GM-CSF antibodies (*n* = 14) versus WT CD4^+^ T cell supernatant plus control antibody (*n* = 14) group. Each dot represents one mammosphere (Mann–Whitney *U* test). **(O)** The number of Ki67^+^ cells per HC11 mammosphere exposed to Il3^KO^ CD4^+^ T cell supernatant plus IL-5/GM-CSF antibodies (*n* = 13) versus WT CD4^+^ T cell supernatant plus control antibody (*n* = 6). Each dot represents one mammosphere (Mann–Whitney *U* test). Bar graph shows mean + SD. All experimental data verified in at least two independent experiments. *, P < 0.05; ***, P < 0.0001.

To determine whether Th2 polarization was required in CD4^+^ T cell immunity against breast carcinogenesis, we examined tumor development in *K14-Tslp*^*tg*^*, MMTV-PyMT*^*tg*^*, Il4r*^*−/−*^ (Tslp-PyMt^tg^ Il4r^KO^) in comparison to Tslp-PyMt^tg^, PyMt^tg^ Il4r^KO^, and PyMt^tg^ animals. Mice that lacked IL-4Rα, and therefore could not mount a Th2 immunity (Fig. S3 A), showed very early breast tumor onset regardless of *Tslp* expression (P = 0.0002 compared with PyMt^tg^ group; Fig. 4 A). The earlier tumor onset did not translate into reduced survival in mice that lacked IL-4Rα, suggesting that the protective role of IL-4 is mainly in the initiation phase of breast tumor development. Tslp-PyMt^tg^ Il4r^KO^ animals developed significantly more breast tumors (P < 0.05 starting at 7 wk of age; Fig. 4 B) and had markedly shorter survival compared with Tslp-PyMt^tg^ mice (P < 0.0001; Fig. 4 C). Loss of IL-4Rα resulted in significantly higher tumor histological grades in Tslp-PyMt^tg^ Il4r^KO^ mice associated with lung metastasis compared with Tslp-PyMt^tg^ mice (P = 0.001; Fig. 4 D and Fig. S3, B–D). To determine whether IL-4/13 signaling mediates the effects of Th2 cells onto breast tumor cells, we implanted PyMt^tg^ or PyMt^tg^ Il4r^KO^ primary breast tumors into the abdominal mammary fat pad of Tslp^tg^ (test) versus WT (control) mice and monitored tumor formation and growth over time. Test mice were protected against breast tumor growth even in the absence of IL-4Rα on the tumor cells (Fig. 4, E and F). Furthermore, we demonstrated that IL4-Rα expression on CD4^+^ T cells was required for the TSLP-induced suppression of PyMt^tg^ breast tumor growth (Fig. S3, E and F). Collectively, these findings demonstrate that Th2 polarization is essential for TSLP-induced CD4^+^ T cell immunity against breast cancer development. However, IL-4 and IL-13 do not directly mediate the tumor-suppressing effect of Th2 cells onto the breast tumor cells.

### Antitumor CD4^+^ T cell response is antigen specific

**Figure 5.**
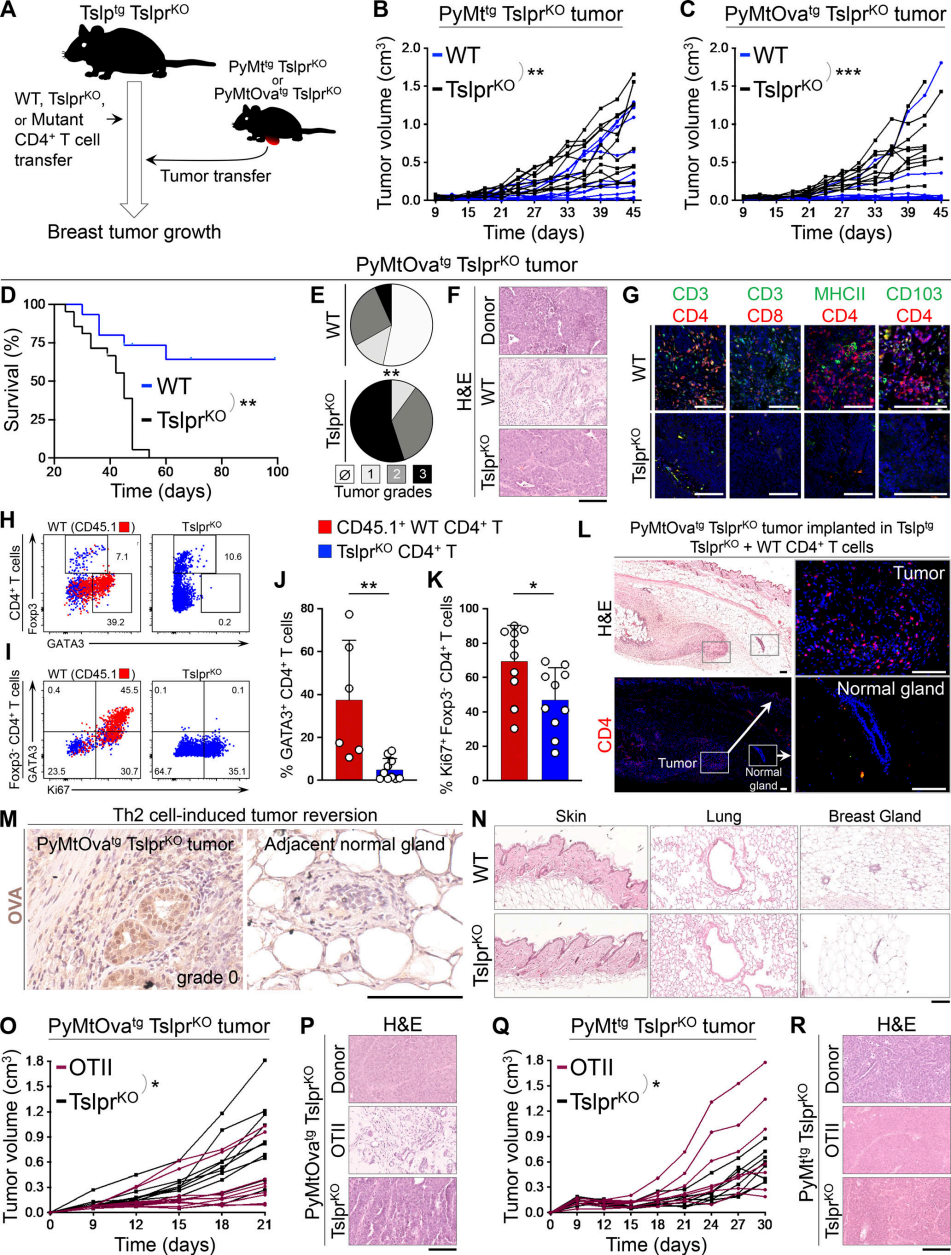
**Antigen specificity of Th2 cell immunity against breast carcinogenesis. (A)** Schematic diagram of the experimental paradigm used to elucidate the effector mechanism of TSLP-stimulated CD4^+^ T cells against breast cancer. Note that only transferred CD4^+^ T cells in the test groups express TSLP receptor (Tslpr), but all other cells including the implanted tumor cells lack TSLP receptor (i.e., Tslpr^KO^). **(B)** Spider plot of PyMt^tg^ Tslpr^KO^ primary breast tumor growth in Tslp^tg^ Tslpr^KO^ mice injected with WT (test, *n* = 10, 6/10 tumors were <0.5 cm^3^ at the endpoint) versus Tslpr^KO^ (control, *n* = 10, 4/10 tumors were <0.5 cm^3^ at the endpoint) CD4^+^ T cells (two-way ANOVA). **(C)** Spider plot of PyMtOva^tg^ Tslpr^KO^ primary tumor growth in Tslp^tg^ Tslpr^KO^ mice injected with WT (*n* = 10, 9/10 tumors were <0.5 cm^3^ at the endpoint) versus Tslpr^KO^ (*n* = 10, 2/10 tumors were <0.5 cm^3^ at the endpoint) CD4^+^ T cells (two-way ANOVA). **(D)** Survival rate of Tslp^tg^ Tslpr^KO^ mice injected with WT (*n* = 15) versus Tslpr^KO^ (*n* = 19) CD4^+^ T cells followed by PyMtOva^tg^ Tslpr^KO^ primary tumor implantation (log-rank test). **(E)** Distribution of histological grades of PyMtOva^tg^ Tslpr^KO^ primary tumors developed in Tslp^tg^ Tslpr^KO^ mice injected with WT (*n* = 15) versus Tslpr^KO^ (*n* = 20) CD4^+^ T cells (Fisher’s exact test). **(F)** Representative images of H&E-stained PyMtOva^tg^ Tslpr^KO^ breast tumors from (top to bottom): tumor donor, Tslp^tg^ Tslpr^KO^ mouse injected with WT CD4^+^ T cells, and mouse injected with Tslpr^KO^ CD4^+^ T cell. Note the degree of breast tumor differentiation in WT (test) group compared with the original tumor harvested from the donor mouse and Tslpr^KO^ (control) group. **(G)** Representative images of CD3/CD4, CD3/CD8, MHCII/CD4, and CD103/CD4 immunofluorescence staining on PyMtOva^tg^ Tslpr^KO^ primary tumors developed in Tslp^tg^ Tslpr^KO^ mice injected with WT versus Tslpr^KO^ CD4^+^ T cells. Note that CD103 colocalizes with CD4 and CD3 on few cells in the WT tumors (scale bars, 100 μm). **(H and I)** Representative flow plots showing percentage GATA3^+^ Th2 and Foxp3^+^ regulatory T cells (Tregs) in PyMtOva^tg^ Tslpr^KO^ breast tumors of Tslp^tg^ Tslpr^KO^ mice injected with CD45.1^+^ WT versus Tslpr^KO^ CD4^+^ T cells (H) and percentage GATA3^+^ and Ki67^+^ cells among tumor-infiltrating Foxp3^−^ CD4^+^ T effector cells in Tslp^tg^ Tslpr^KO^ mice injected with WT or Tslpr^KO^ CD4^+^ T cells (I). Injected CD45.1^+^ WT CD4^+^ T cells are highlighted in red; Tslpr^KO^ CD4^+^ T cells are in blue. Numbers on the plots represent the percentage of cells within each gate. **(J and K)** Percentage GATA3^+^ Th2 cells among CD45.1^+^ WT CD4^+^ T cells (test, *n* = 6) versus Tslpr^KO^ CD4^+^ T cells (control, *n* = 10; J) and Ki67^+^ cells among CD45.1^+^ WT Foxp3^−^ CD4^+^ T cells (test, *n* = 10) versus Tslpr^KO^ Foxp3^−^ CD4^+^ T cells (control, *n* = 10; K) isolated from PyMtOva^tg^ Tslpr^KO^ breast tumors developed in Tslp^tg^ Tslpr^KO^ mice (Mann-Whitney *U* test). Bar graphs show mean + SD. **(L)** H&E and immunofluorescence images show breast tumor and adjacent mammary gland of a Tslp^tg^ Tslpr^KO^ mouse injected with WT CD4^+^ T cells and implanted with PyMtOva^tg^ Tslpr^KO^ tumor. CD4 immunofluorescence staining highlights CD4^+^ T cells infiltrating the tumor, but not the adjacent normal mammary gland of the mouse (insets: scale bars, 100 μm). **(M)** Representative images of immunohistochemical staining for OVA in breast tumor and adjacent mammary gland of Tslp^tg^ Tslpr^KO^ mice implanted with high-grade PyMtOva^tg^ Tslpr^KO^ tumor. Note that CD4^+^ Th2 cell immunity suppressed an OVA-expressing tumor by transforming it to gland-like structures while sparing the OVA-negative adjacent mammary glands (scale bar: 100 μm). **(N)** Representative images of H&E-stained skin, lung, and mammary glands of Tslp^tg^ Tslpr^KO^ mice injected with WT versus Tslpr^KO^ CD4^+^ T cells and implanted with PyMtOva^tg^ Tslpr^KO^ tumor. No sign of inflammation is detected in the nontumor tissues at the endpoint (scale bar: 100 μm). **(O)** Spider plot showing PyMtOva^tg^ Tslpr^KO^ primary tumor growth in Tslp^tg^ Tslpr^KO^ mice injected with OTII (*n* = 9, 7/9 tumors were <0.5 cm^3^ at the endpoint) versus Tslpr^KO^ (*n* = 10, 2/10 tumors were <0.5 cm^3^ at the endpoint) CD4^+^ T cells (two-way ANOVA). **(P)** Representative images of H&E-stained PyMtOva^tg^ Tslpr^KO^ breast tumors from (top to bottom): tumor donor, tumor recipient mice injected with OTII, or Tslpr^KO^ CD4^+^ T cells (scale bar: 100 μm). **(Q)** Spider plot of PyMt^tg^ Tslpr^KO^ primary tumor growth in Tslp^tg^ Tslpr^KO^ mice injected with Ova-specific CD4^+^ T cells (OTII, *n* = 7, 3/7 tumors were <0.5 cm^3^ at the endpoint) versus Tslpr^KO^ CD4^+^ T cells (*n* = 8, 3/8 tumors were <0.5 cm^3^ at the endpoint, two-way ANOVA). **(R)** Representative images of H&E-stained PyMt^tg^ Tslpr^KO^ breast tumors from (top to bottom): tumor donor mouse, mouse injected with OTII CD4^+^ T cells, and mouse injected with Tslpr^KO^ CD4^+^ T cells (scale bar: 100 μm). Each of the tumors in the studies is from a separate mouse. All experimental data verified in at least two independent experiments. *, P < 0.05; **, P < 0.01; ***, P < 0.0001.

To determine the mechanism of Th2 cell immunity against breast carcinogenesis, we designed an experimental paradigm in which Tslp^tg^ Tslpr^KO^ mice on the C57BL/6 background received naive Tslpr^KO^ (control), WT, or mutant CD4^+^ T cells. Mutant CD4^+^ T cells included OTII, Il4^KO^, Tnf^KO^, Ifn^KO^, and Il3^KO^. 1 d after adoptive T cell transfer, primary breast tumors from PyMt^tg^ Tslpr^KO^ or *MMTV-PyMT-mCherry-Ova*^*tg*^*, Tslpr*^*−/−*^ mice (PyMtOva^tg^ Tslpr^KO^, expressing OVA in tumor cells; Engelhardt et al., 2012) on the C57BL/6 background were implanted into the abdominal mammary fat pad of recipient Tslp^tg^ Tslpr^KO^ mice, which were then monitored for breast tumor growth over time (Fig. 5 A). PyMt^tg^ Tslpr^KO^ primary breast tumor cells generated significantly smaller tumors in Tslp^tg^ Tslpr^KO^ mice that received WT CD4^+^ T cells compared with Tslpr^KO^ CD4^+^ T cells (P = 0.0087; Fig. 5 B). The breast cancer suppression in the Tslp^tg^ Tslpr^KO^ + WT CD4^+^ T cell group was more pronounced when the tumor cells expressed OVA antigen (PyMtOva^tg^ Tslpr^KO^, P < 0.0001; Fig. 5 C). Enhanced protection against PyMtOva^tg^ Tslpr^KO^ tumors in mice that received WT CD4^+^ T cells resulted in long-term survival of 67% of these animals (P = 0.0002, Fig. 5 D). Although the implanted PyMtOva^tg^ Tslpr^KO^ tumor (donor tumor) had a high histological grade, recipient mice in the Tslp^tg^ Tslpr^KO^ + WT CD4^+^ T cell (test) group developed low-grade tumors (P = 0.0005, Fig. 5, E and F). The majority of Tslp^tg^ Tslpr^KO^ mice with WT CD4^+^ T cells either did not have a detectable tumor focus on the histology or developed cystic structures surrounded by fibrotic tissue at the site of implanted tumor at the completion of the study, which corresponded to the lack of tumor growth and long-term survival of these animals (Fig. 5 F). Breast cancer suppression in the test group was associated with large CD4^+^ T and few CD103^+^ CD4^+^ T_RM_ cell infiltrates surrounding the low-grade tumors (Fig. 5 G). Transferring congenically marked (CD45.1^+^) WT CD4^+^ T cells into Tslp^tg^ Tslpr^KO^ mice revealed that the majority of these cells became GATA3^+^ Th2 cells, which actively proliferated in PyMtOva^tg^ Tslpr^KO^ tumors (Fig. 5, H–K). CD4^+^ T cell immunity against OVA-positive tumor cells did not affect the nearby normal mammary glands (Fig. 5, L and M). Likewise, TSLP-stimulated WT CD4^+^ T cells in Tslp^tg^ Tslpr^KO^ mice did not cause inflammation in skin, lung, or normal mammary glands (Fig. 5 N). These results demonstrate that CD4^+^ Th2 cells drive the differentiation of advanced malignant cells and prevent the progression of breast tumors to a high-grade state.

Next, we investigated the antigen specificity of the TSLP-stimulated Th2 cell responses by transferring either OVA-specific CD4^+^ T cells from OTII Rag2^KO^ mice (test; Barnden et al., 1998) or Tslpr^KO^ CD4^+^ T cells (control) into Tslp^tg^ Tslpr^KO^ mice that were then implanted with primary PyMtOva^tg^ Tslpr^KO^ or PyMt Tslpr^KO^ tumor cells. As expected, OTII T cells protected Tslp^tg^ Tslpr^KO^ mice from PyMtOva^tg^ Tslpr^KO^ tumor development (P = 0.0107; Fig. 5 O). OTII T cells reverted high-grade PyMtOva^tg^ Tslpr^KO^ donor tumor into low-grade fibrocystic structures in the recipient Tslp^tg^ Tslpr^KO^ animals (Fig. 5 P). In contrast, OTII T cells were not able to protect Tslp^tg^ Tslpr^KO^ mice from PyMt^tg^ Tslpr^KO^ tumors, which lacked OVA expression and grew larger in the presence of OTII compared with Tslpr^KO^ T cells (P = 0.0216; Fig. 5, Q and R). This finding demonstrates that TSLP-induced Th2 immunity in the breast is antigen specific.

### Th2 cell immunity is mediated by IL-3, IL-5, and GM-CSF effector cytokines

**Figure 6.**
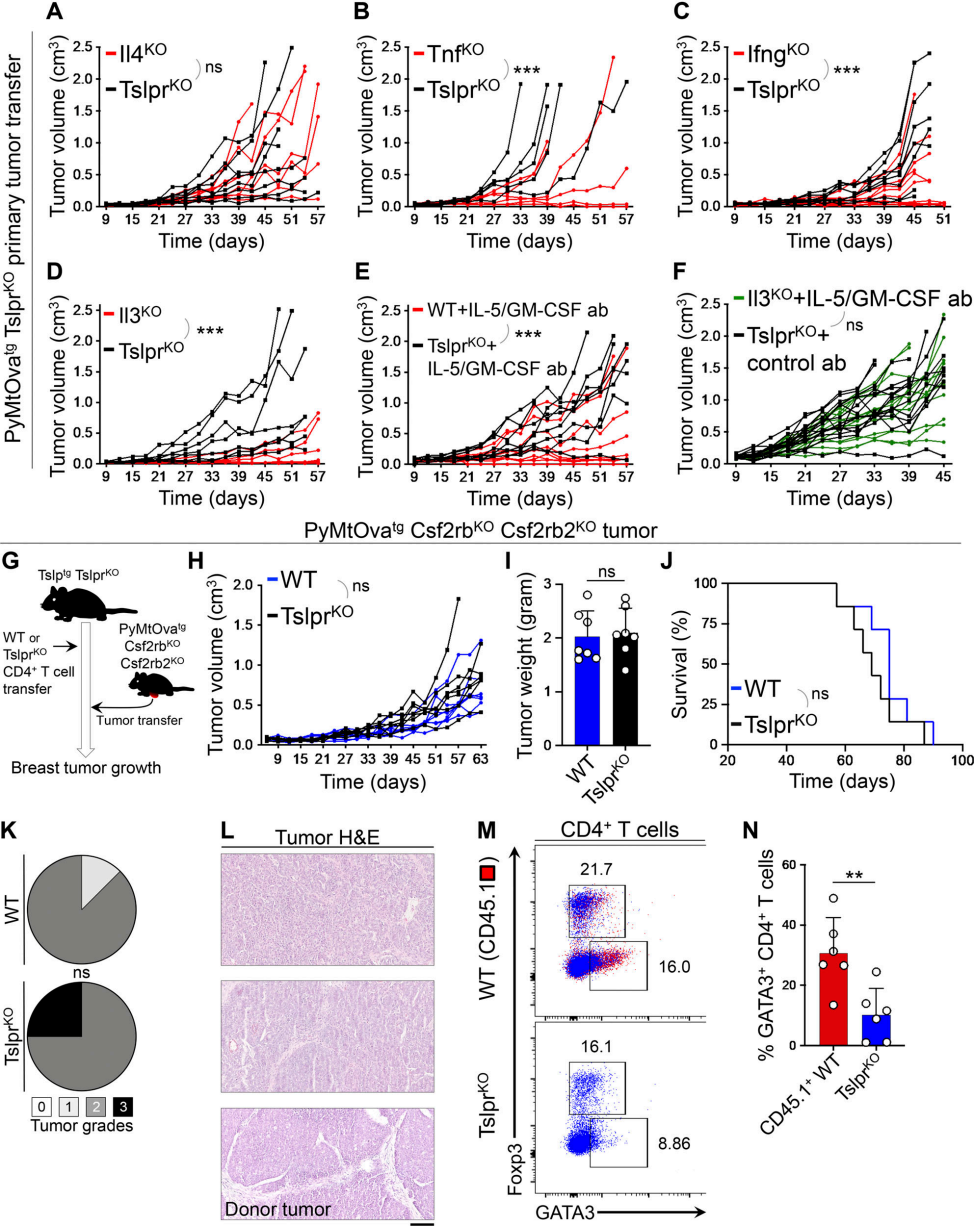
**IL-3, IL-5, and GM-CSF cytokines mediate Th2 cell immunity against breast carcinogenesis. (A–C)** Spider plots of PyMtOva^tg^ Tslpr^KO^ primary tumor growth in Tslp^tg^ Tslpr^KO^ mice injected with Il4^KO^ (*n* = 8, 2/8 tumors were <0.5 cm^3^ at the endpoint; A), Tnf^KO^ (*n* = 6, 2/6 tumors were <0.5 cm^3^ at the endpoint; B), Ifng^KO^ (*n* = 10, 6/10 tumors were <0.5 cm^3^ at the endpoint; C) mutant CD4^+^ T cells compared to Tslpr^KO^ CD4^+^ T cells (*n* = 8, 3/8 tumors were <0.5 cm^3^; *n* = 5, 0/5 tumors were <0.5 cm^3^; and *n* = 8, 2/8 tumors were <0.5 cm^3^ at the endpoint, respectively; two-way ANOVA). **(D and E)** Spider plots of primary PyMtOva^tg^ Tslpr^KO^ tumor growth in Tslp^tg^ Tslpr^KO^ mice injected with Il3^KO^ (*n* = 6, 4/6 tumors were <0.5 cm^3^ at the endpoint) versus Tslpr^KO^ (*n* = 7, 2/7 tumors were <0.5 cm^3^ at the endpoint) CD4^+^ T cells (two-way ANOVA; D) and WT (*n* = 9, 6/9 tumors were <0.5 cm^3^ at the endpoint) versus Tslpr^KO^ (*n* = 8, 1/8 tumors were <0.5 cm^3^ at the endpoint) CD4^+^ T cells (E) while both groups were treated with anti–IL-5 plus anti–GM-CSF blocking antibodies (ab; two-way ANOVA). **(F)** Spider plot of PyMtOva^tg^ Tslpr^KO^ primary tumor growth in Tslp^tg^ Tslpr^KO^ mice injected with Il3^KO^ CD4^+^ T cells in combination with anti–IL-5/GM-CSF antibodies (*n* = 13, 1/13 tumors were <0.5 cm^3^ at the endpoint) versus Tslpr^KO^ CD4^+^ T cells and rat IgG isotype control antibody (*n* = 14, 1/14 tumors were <0.5 cm^3^ at the endpoint; two-way ANOVA). **(G)** Schematic diagram of the experimental paradigm used to elucidate the role of IL-3/IL-5/GM-CSF receptors on breast cancer cells in mediating the antitumor effects of TSLP-stimulated CD4^+^ T cells against breast cancer. **(H)** Spider plot of PyMtOva^tg^ Csf2rb^KO^ Csf2rb2^KO^ primary tumor growth in Tslp^tg^ Tslpr^KO^ mice injected with WT (*n* = 7, 1/7 tumors were <0.5 cm^3^ at the endpoint) versus Tslpr^KO^ CD4^+^ T cells (*n* = 7, 1/7 tumors were <0.5 cm^3^ at the endpoint, two-way ANOVA). **(I)** PyMtOva^tg^ Csf2rb^KO^ Csf2rb2^KO^ tumor weights at the endpoint in Tslp^tg^ Tslpr^KO^ mice injected with WT (*n* = 7) versus Tslpr^KO^ (*n* = 7) CD4^+^ T cells (Mann-Whitney *U* test). **(J)** Percentage survival of Tslp^tg^ Tslpr^KO^ mice injected with WT (*n* = 7) versus Tslpr^KO^ (*n* = 7) CD4^+^ T cells followed by PyMtOva^tg^ Csf2rb^KO^ Csf2rb2^KO^ primary tumor implantation (log-rank test). **(K)** Distribution of histological grades of PyMtOva^tg^ Csf2rb^KO^ Csf2rb2^KO^ primary tumors developed in Tslp^tg^ Tslpr^KO^ mice injected with WT (*n* = 7) versus Tslpr^KO^ (*n* = 7) CD4^+^ T cells (Fisher’s exact test). **(L)** Representative images of H&E-stained PyMtOva^tg^ Csf2rb^KO^ Csf2rb2^KO^ breast tumors from Tslp^tg^ Tslpr^KO^ mouse injected with WT CD4^+^ T cells, Tslp^tg^ Tslpr^KO^ mouse injected with Tslpr^KO^ CD4^+^ T cells, and the donor tumor from PyMtOva^tg^ Csf2rb^KO^ Csf2rb2^KO^ mouse (scale bar: 100 μm). **(M)** Representative flow plots of transcription factor expression in PyMtOva^tg^ Csf2rb^KO^ Csf2rb2^KO^ breast tumor–infiltrating CD4^+^ T cells isolated from Tslp^tg^ Tslpr^KO^ mice injected with CD45.1^+^ WT versus TSLPR^KO^ CD4^+^ T cells. Numbers on the plots represent the percentage of cells within each gate. **(N)** Percentage of GATA3^+^ Th2 cells among CD45.1^+^ WT CD4^+^ T cells (test, *n* = 6) versus Tslpr^KO^ CD4^+^ T cells (control, *n* = 6) isolated from PyMtOva^tg^ Csf2rb^KO^ Csf2rb2^KO^ breast tumors in Tslp^tg^ Tslpr^KO^ mice (Mann–Whitney *U* test). Bar graph shows mean + SD. Each of the tumors in the studies is from a separate mouse. All experimental data verified in at least two independent experiments. *, P < 0.05; **, P < 0.01; ***, P < 0.0001.

To determine which factors released by TSLP-stimulated Th2 cells are responsible for breast cancer suppression, we studied mutant CD4^+^ T cells that lacked either cytokines commonly associated with antitumor immunity (TNFα and IFNγ) or those that were highly expressed by TSLP-stimulated CD4^+^ Th2 cells (IL-3, IL-5, and GM-CSF). In these experiments, Tslp^tg^ Tslpr^KO^ mice were injected with either cytokine-deficient or Tslpr^KO^ CD4^+^ T cells followed by PyMtOva^tg^ Tslpr^KO^ tumor implantation (Fig. 5 A). Consistent with their defect in Th2 polarization, Il4^KO^ CD4^+^ T cells were not able to protect Tslp^tg^ Tslpr^KO^ mice against PyMtOva^tg^ Tslpr^KO^ tumor growth (Figs. 6 A and S3 G). In contrast, Tnf^KO^ and Ifng^KO^ CD4^+^ T cells blocked PyMtOva^tg^ Tslpr^KO^ breast tumor growth and induced the differentiation of the high-grade donor tumors into low-grade fibrocystic structures in Tslp^tg^ Tslpr^KO^ mice (Fig. 6, B and C; and Fig. S3, H–J). Next, we examined the effector function of IL-3 in Th2 cell immunity against breast cancer. Il3^KO^ CD4^+^ T cells suppressed PyMtOva^tg^ Tslpr^KO^ breast tumor growth in Tslp^tg^ Tslpr^KO^ mice (Figs. 6 D and S3 K). Likewise, treatment with the combination of IL-5 and GM-CSF blocking antibodies did not reverse the tumor-suppressing function of WT CD4^+^ T cells in Tslp^tg^ Tslpr^KO^ mice (Figs. 6 E and S3 L).

The receptors for IL-3, IL-5, and GM-CSF share a common β chain (CSF2RB or CD131), which is the signaling arm of these receptors (Broughton et al., 2012). To determine whether a simultaneous blockade of IL-3, IL-5, and GM-CSF can inhibit the antitumor effect of TSLP-stimulated CD4^+^ T cells, we used our mammosphere/T cell supernatant culture system in which naive CD4^+^ T cells from spleen of Il3^KO^ and WT mice were stimulated ex vivo using anti-CD3/CD28 antibodies plus TSLP over one cycle. HC11 cells in the 3D mammosphere culture system were exposed to the supernatants from Il3^KO^ versus WT T cells together with IL-5– and GM-CSF–blocking antibodies versus IgG control antibody, respectively. Mammospheres cultured in the supernatant from Il3^KO^ CD4^+^ T cells plus anti–IL-5/GM-CSF antibodies (test) formed significantly larger mammospheres with higher proliferation compared with those cultured in WT CD4^+^ T cell supernatant plus IgG (control; Fig. S3, M–O). These results indicate that IL-3, IL-5, and GM-CSF mediate the direct antitumor effects of TSLP-stimulated CD4^+^ T cells on mammary epithelial cells.

**Figure S4.**
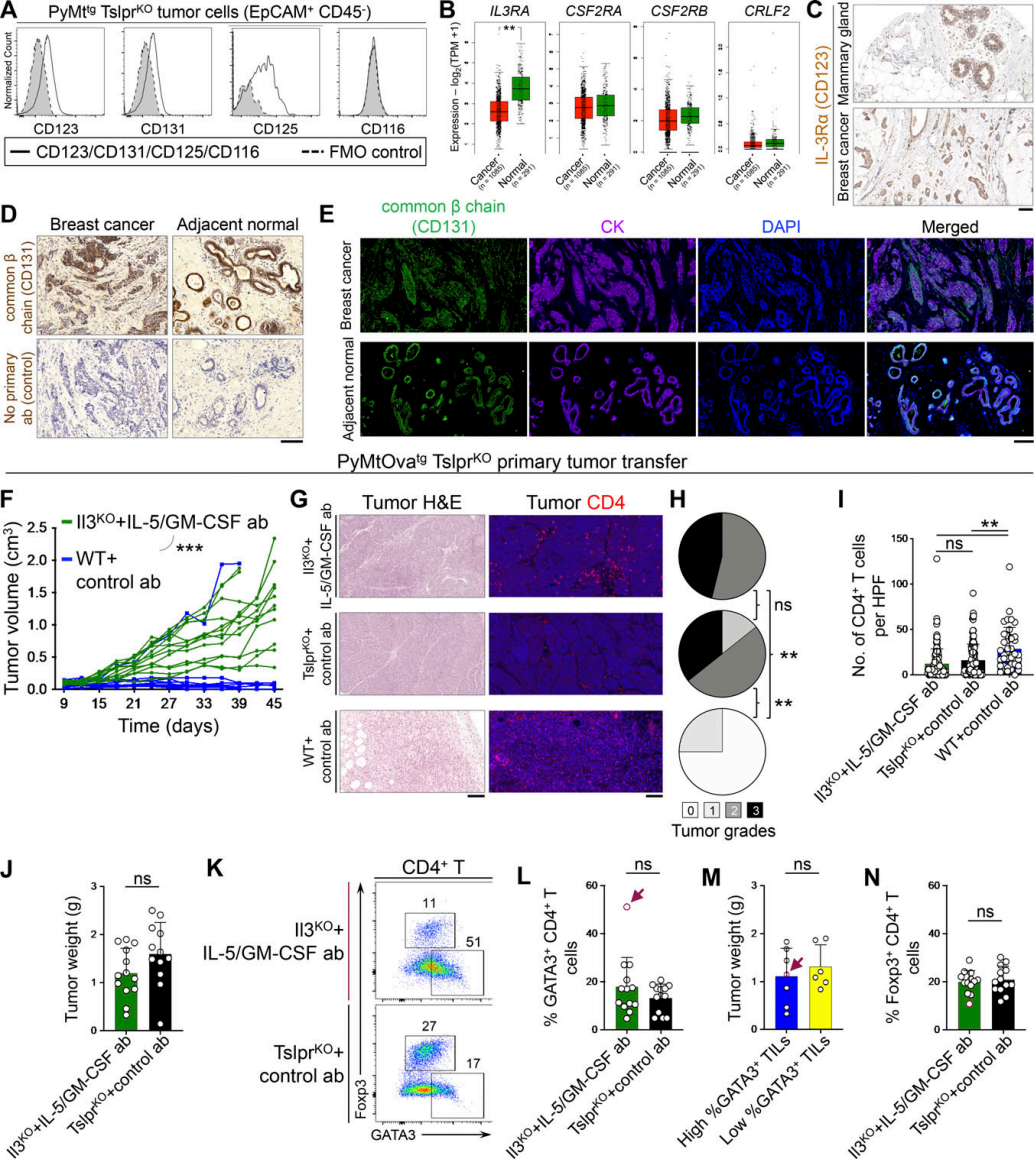
**IL-3, IL-5, and GM-CSF receptors expression and function in breast normal epithelial and cancer cells. (A)** Histogram showing expression levels of mouse IL-3Rα (CD123), common β chain receptor (CD131), IL-5Rα (CD125), and GM-CSFRα (CD116) on the surface of EpCAM^+^ CD45^−^ PyMt^tg^ tumor cells. Gray histograms with dashed outline show fluorescence minus one (FMO) control. **(B)** Box plots of human *IL3RA* (IL-3 receptor α chain)*, CSF2RA* (GM-CSF receptor α chain), *CSF2RB* (common β chain receptor), and *CRLF2* (TSLPR) gene expression in normal mammary glands versus breast cancer samples across TCGA/GTEx datasets (one-way ANOVA, Gene Expression Profiling Interactive Analysis database). **(C)** IL-3Rα immunohistochemical stain of human mammary gland and breast tumor. Images were obtained from the Human Protein Atlas website (https://v18.proteinatlas.org). **(D)** Representative images of common β chain receptor (CD131) IHC staining on human breast tumor and adjacent normal breast glands. Control staining with no primary antibody is also shown. **(E)** Representative images of common β chain receptor (CD131) and cytokeratin (CK) immunofluorescence staining on human breast tumor and adjacent normal breast glands. **(F)** Spider plot of PyMtOva^tg^ Tslpr^KO^ primary tumor growth in Tslp^tg^ Tslpr^KO^ mice injected with Il3^KO^ CD4^+^ T cells in combination with anti–IL-5/GM-CSF antibodies (*n* = 13, 1/13 tumors were <0.5 cm^3^ at the endpoint) versus WT CD4^+^ T cells and Rat IgG isotype control antibody (*n* = 10, 9/10 tumors were <0.5 cm^3^ at the endpoint; two-way ANOVA). Note that Il3^KO^ CD4^+^ T cells plus anti–IL-5/GM-CSF antibodies tumor growth data are also shown in Fig. 6 F. **(G)** Representative H&E and CD4-immunostained images of PyMtOva^tg^ Tslpr^KO^ primary tumors developed in Tslp^tg^ Tslpr^KO^ mice injected with Il3^KO^ CD4^+^ T cells plus anti–IL-5 and anti–GM-CSF blocking antibodies (test) versus Tslpr^KO^ CD4^+^ T cells plus isotype control antibody (negative control) and WT CD4^+^ T cells plus isotype control antibody (positive control). **(H)** Distribution of histological grades of PyMtOva^tg^ Tslpr^KO^ primary tumors developed in Tslp^tg^ Tslpr^KO^ mice injected with Il3^KO^ CD4^+^ T cells plus anti–IL-5 and anti–GM-CSF blocking antibodies (test, *n* = 13) versus Tslpr^KO^ CD4^+^ T cells plus isotype control antibodies (negative control, *n* = 14) and WT CD4^+^ T cells plus isotype control antibodies (positive control, *n* = 4, Fisher’s exact test). **(I)** Quantification of CD4^+^ T cells in PyMtOva^tg^ Tslpr^KO^ primary tumors developed in Tslp^tg^ Tslpr^KO^ mice injected with Il3^KO^ CD4^+^ T cells plus anti–IL-5 and anti–GM-CSF blocking antibodies (test, *n* = 13) versus Tslpr^KO^ CD4^+^ T cells plus isotype control antibody (negative control, *n* = 12) and WT CD4^+^ T cells plus isotype control antibody (positive control, *n* = 4). CD3/CD4 double-positive cells were counted in 10 HPF images per tumor sample. HPF images for each sample were chosen randomly across the tumor section. Each dot represents one HPF image (unpaired *t* test). **(J)** PyMtOva^tg^ Tslpr^KO^ tumor weight developed in Tslp^tg^ Tslpr^KO^ mice injected with Il3^KO^ CD4^+^ T cells plus anti–IL-5 and anti–GM-CSF blocking antibodies (*n* = 13) versus Tslpr^KO^ CD4^+^ T cells plus isotype control antibody (*n* = 12). Note that WT CD4^+^ T cells plus isotype control antibody (positive control) were not included in this analysis, as only one of four tumors had an appreciable mass (Mann–Whitney *U* test). **(K)** Flow plot showing percentage GATA3^+^ Th2 and Foxp3^+^ regulatory T cells (Tregs) among tumor-infiltrating CD4^+^ T cells in Il3^KO^ CD4^+^ T cells plus anti–IL-5 and anti–GM-CSF blocking antibodies versus Tslpr^KO^ CD4^+^ T cells plus isotype control antibody group. **(L)** Percentage GATA3^+^ Th2 cells among tumor-infiltrating CD4^+^ T cells in Il3^KO^ CD4^+^ T cells plus anti–IL-5 and anti–GM-CSF blocking antibodies (*n* = 13) versus Tslpr^KO^ CD4^+^ T cells plus isotype control antibody (*n* = 13) group (Mann–Whitney *U* test). **(M)** Tumor weights compared between the tumors with high (*n* = 7) versus low (*n* = 6) percentage GATA3^+^ Th2 cells in Il3^KO^ CD4^+^ T cells plus anti–IL-5 and anti–GM-CSF blocking antibodies (test) group. Red arrows point to the tumor with the highest percentage of tumor-infiltrating Th2 cells (Mann–Whitney *U* test). **(N)** Percentage Foxp3^+^ Tregs among tumor-infiltrating CD4^+^ T cells in Il3^KO^ CD4^+^ T cells plus anti–IL-5 and anti–GM-CSF blocking antibodies (*n* = 13) versus Tslpr^KO^ CD4^+^ T cells plus isotype control antibody (*n* = 13) group (Mann–Whitney *U* test). Scale bars 100 μm. Bar graph shows mean + SD. All experimental data verified in at least two independent experiments. **, P < 0.01; ***, P < 0.0001.

Common β chain (CD131), IL-3Rα (CD123), and IL-5Rα (CD125) were detectable on the surface of the breast tumor cells in mice (Fig. S4 A). IL-3 and GM-CSF receptor genes (*IL3RA*, *CSF2RA*, and *CSF2RB*) were expressed in human normal mammary epithelial and breast tumor cells at markedly higher levels compared with TSLP receptor (*CRLF2*; Fig. S4 B). IL-3Rα and common β chain proteins were expressed in human normal mammary epithelial and breast tumor cells (Fig. S4, C–E). Importantly, the blockade of all three cytokines by using Il3^KO^ CD4^+^ T cells in combination with IL-5 and GM-CSF blocking antibodies led to accelerated PyMtOva^tg^ Tslpr^KO^ breast tumor growth and formation of high-grade tumors in Tslp^tg^ Tslpr^KO^ mice, similar to the animals that received Tslpr^KO^ CD4^+^ T cells (Fig. 6 F and Fig. S4, F–J). Interestingly, a subset of tumors that contained high Th2 cell infiltrates in the IL-3/IL-5/GM-CSF–deficient group grew as large as tumors with low Th2 cell infiltrates in the test and control groups (Fig. S4, K–N). This finding supports the role of IL-3, IL-5, and GM-CSF as effector cytokines released by Th2 cells to specifically block breast tumor cell proliferation.

To determine whether IL-3, IL-5, and GM-CSF direct signaling to breast tumor cells is the mediator of Th2 cell immunity against breast cancer, we generated *MMTV-PyMT-mCherry-Ova*^*tg*^, *Csf2rb*^*−/−*^, *Csf2rb2*^*−/−*^ (PyMtOva^tg^ Csf2rb^KO^ Csf2rb2^KO^) mice, which develop breast tumors lacking common β chain (*Csf2rb*) and IL-3 receptor class 2 subunit β (*Csf2rb2*) receptors. We implanted primary breast tumors from PyMtOva^tg^ Csf2rb^KO^ Csf2rb2^KO^ mice into the abdominal mammary fat pad of Tslp^tg^ Tslpr^KO^ recipient mice a day after adoptive WT CD4^+^ T cell (test) versus Tslpr^KO^ CD4^+^ T cell (control) transfer into these animals. Recipient mice were monitored for breast tumor growth over time (Fig. 6 G). Although the transferred CD45.1^+^ WT CD4^+^ T cells acquired Th2 phenotype in PyMtOva^tg^ Csf2rb^KO^ Csf2rb2^KO^ tumors, they failed to suppress tumor growth, extend animals’ survival, or promote tumor differentiation compared with Tslpr^KO^ CD4^+^ T cells in Tslp^tg^ Tslpr^KO^ mice (Fig. 6, H–N). These findings demonstrate that IL-3/IL-5/GM-CSF receptors on breast tumor cells are required for the antitumor effects of TSLP-activated CD4^+^ T cells.

### Th2 cell immunity is durable and reverts high-grade breast tumors into low-grade fibrocystic structures

**Figure S5.**
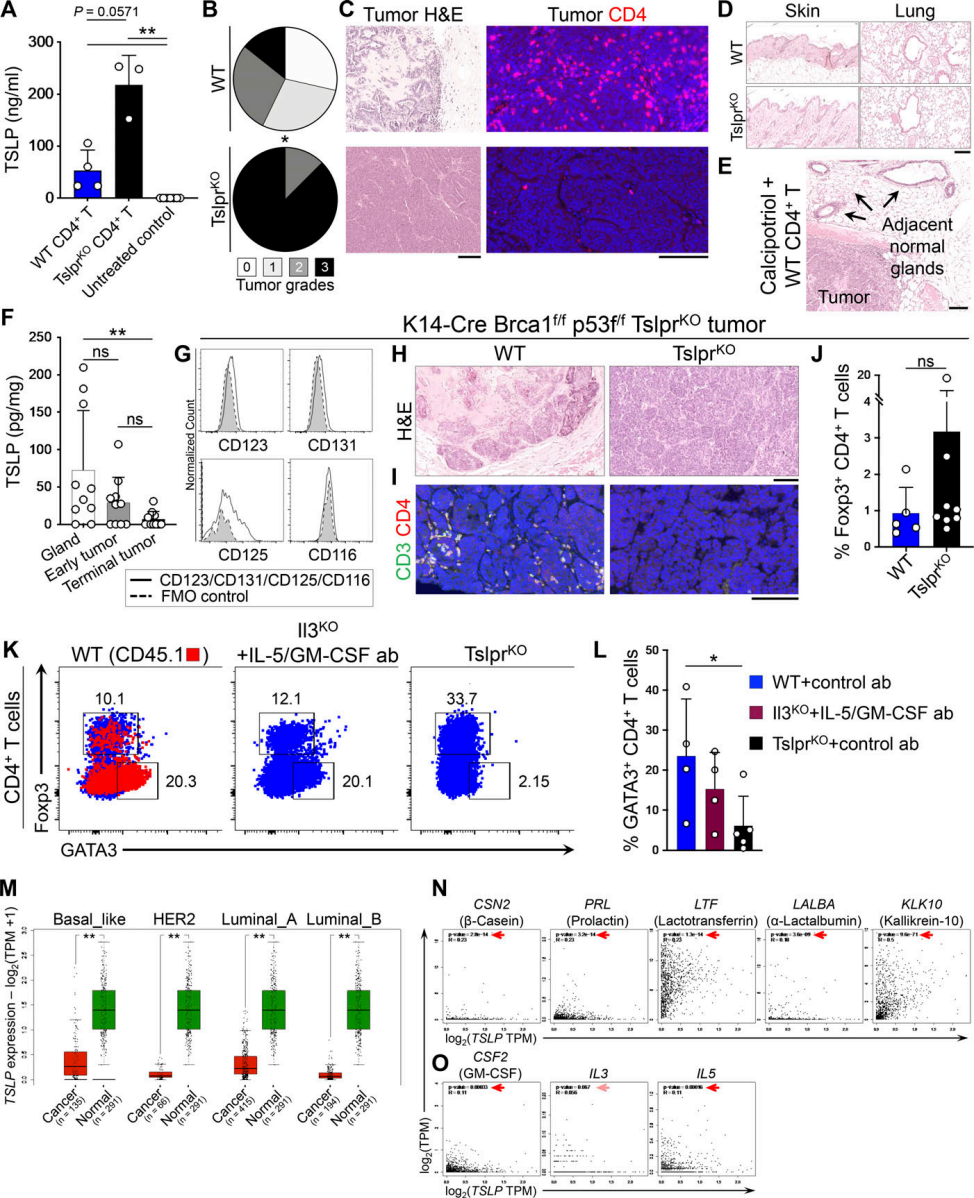
**Topical TSLP induction and Th2 cell immunity against mouse and human breast cancer. (A)** TSLP protein levels measured with ELISA in plasma of Tslpr^KO^ mice transferred with PyMtOva^tg^ Tslpr^KO^ primary tumors and WT CD4^+^ T cells (*n* = 4) or TslprKO CD4^+^ T cells (*n* = 3) 24 h after topical calcipotriol application. Plasma from untreated WT mice (*n* = 7) was used as controls (bar graph shows mean + SD, Mann–Whitney *U* test). **(B)** Distribution of histological grades of PyMtOva^tg^ Tslpr^KO^ primary tumors developed in Tslpr^KO^ mice injected with WT (*n* = 7) versus Tslpr^KO^ (*n* = 8) CD4^+^ T cells and treated with topical calcipotriol (Fisher’s exact test). **(C)** Representative H&E and CD4-immunostained images of PyMtOva^tg^ Tslpr^KO^ primary tumors developed in Tslpr^KO^ mice treated with calcipotriol and injected with WT or Tslpr^KO^ CD4^+^ T cells. **(D)** Representative images of H&E-stained skin and lung tissue from Tslpr^KO^ mice injected with WT or Tslpr^KO^ CD4^+^ T cells and implanted with PyMtOva^tg^ Tslpr^KO^ after topical calcipotriol treatment. Note the absence of inflammation in the normal barrier organs of the mice. **(E)** Representative images of H&E-stained breast tumor and adjacent mammary glands of Tslpr^KO^ mice treated with topical calcipotriol after receiving WT CD4^+^ T cells and PyMtOva^tg^ Tslpr^KO^ primary tumor transfer. Note the absence of inflammation around the normal mammary glands of the mouse. **(F)** TSLP protein levels in tissue lysates from WT breast glands, early breast tumors (postnatal day 60–90), and terminal breast cancers from PyMt^tg^ mice on the BALB/c background (Mann–Whitney *U* test). **(G)** Histogram showing expression levels of mouse IL-3Rα (CD123), common β chain receptor (CD131), IL-5Rα (CD125), and GM-CSFRα (CD116) on the surface of EpCAM^+^ CD45^−^ K14-Cre Brca1^f/f^ p53f^/f^ Tslpr^KO^ tumor cells. Gray histograms with dashed outline show FMO control. **(H and I)** Representative images of H&E-stained (H) and CD3/CD4-stained (I) K14-Cre Brca1^f/f^ p53f^/f^ Tslpr^KO^ tumors developed in Rag1^KO^ mice injected with WT or Tslpr^KO^ CD4^+^ T cells and treated with topical calcipotriol. **(J)** Percentage Foxp3^+^ Tregs among WT CD4^+^ T cells (test, *n* = 5) versus Tslpr^KO^ CD4^+^ T cells (control, *n* = 8) isolated from K14-Cre Brca1^f/f^ p53f^/f^ Tslpr^KO^ breast tumors grown in Rag1^KO^ mice (Mann–Whitney *U* test). **(K)** Representative flow plots of transcriptional factor expression in CD4^+^ T cells isolated from P48-Cre LSL-Kras^G12D^ p53^f/f^ pancreatic tumor from Tslp^tg^ Tslpr^KO^ mice injected with CD45.1^+^ WT, Il3^KO^ plus IL-5/GM-CSF antibodies or TSLPR^KO^ CD4^+^ T cells. Numbers on the plots represent the percentage of cells within each gate. **(L)** Percentage GATA3^+^ Th2 cells among CD45.1^+^ WT CD4^+^ T cells (*n* = 4), Il3^KO^ plus IL-5/GM-CSF antibodies (*n* = 4), and Tslpr^KO^ CD4^+^ T cells (*n* = 5) isolated from P48-Cre LSL-Kras^G12D^ p53^f/f^ pancreatic tumors (Mann–Whitney *U* test). **(M)** Box plots of *TSLP* expression in normal mammary glands versus different breast cancer subtypes across TCGA/GTEx datasets (one-way ANOVA, Gene Expression Profiling Interactive Analysis database). **(N)** Correlation between *TSLP* and differentiation genes expression in breast cancers in TCGA. Note that *CSN2* (β-casein), *PRL* (prolactin), *LTF* (Lactotransferrin), *LALBA* (α-lactalbumin), and *KLK10* (kallikrein-10) are breast gland differentiation genes that were found to be most upregulated in *Tslp* overexpressing PyMt^tg^ tumors. **(O)** Correlation between *TSLP* and *CSF2*/*IL3*/*IL5* in breast cancers in TCGA. Significant correlations are highlighted by red arrows (Spearman’s rank correlation, Gene Expression Profiling Interactive Analysis database). Scale bars 100 μm. Bar graph shows mean + SD. All murine experimental data verified in at least two independent experiments. *, P < 0.05; **, P < 0.01.

**Figure 7.**
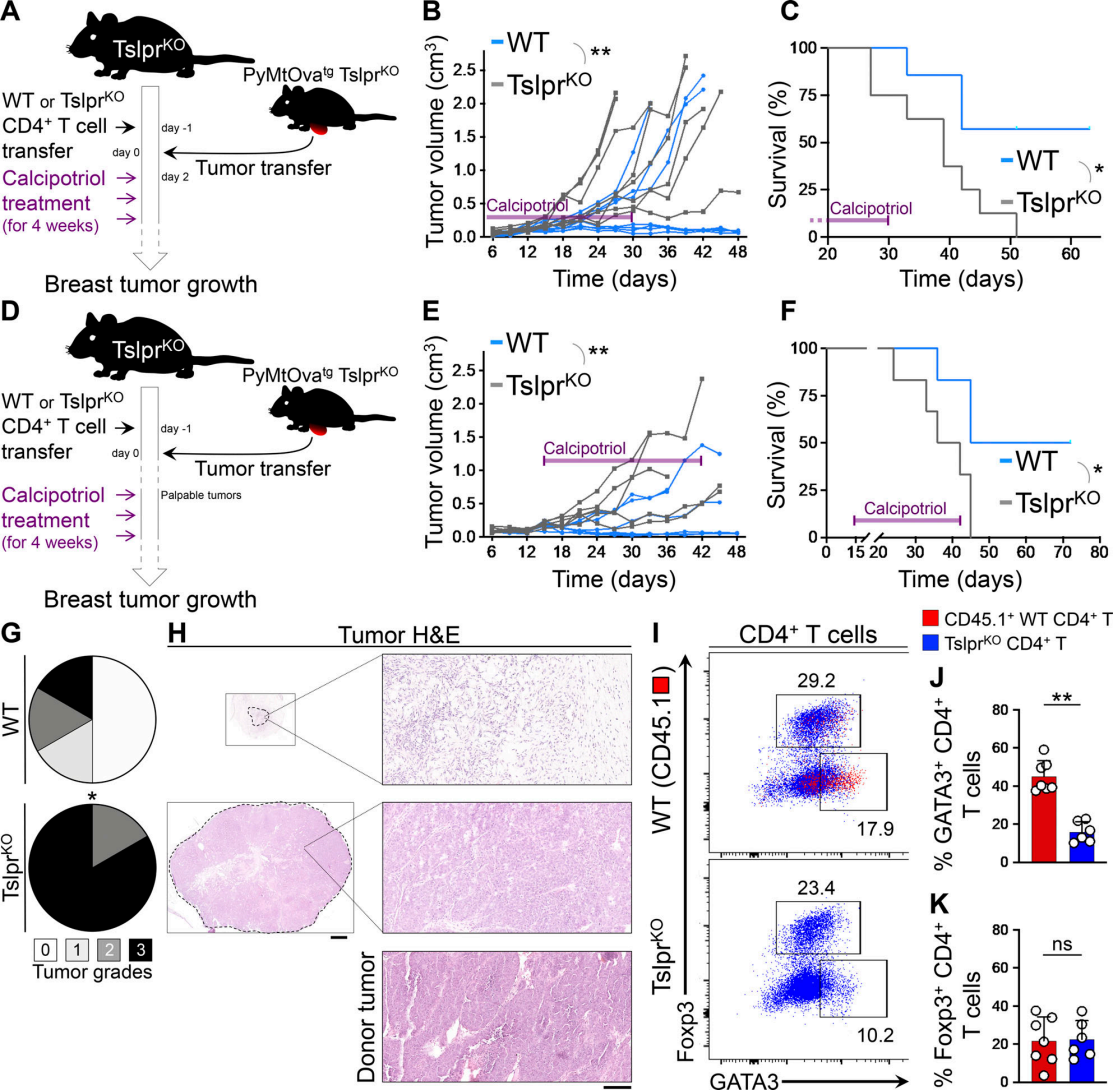
**Transient induction of TSLP provides lasting immunity against breast cancer. (A)** Schematic diagram of the experimental procedure used to test the effect of transient topical TSLP induction on Th2 cell immunity against primary breast tumor growth. **(B and C)** Tslpr^KO^ mice received WT or Tslpr^KO^ CD4^+^ T cells, PyMtOva^tg^ Tslpr^KO^ breast tumor, and topical calcipotriol treatment. Tumor growth kinetics (two-way ANOVA; B) and survival rate (log-rank test; C) of Tslpr^KO^ mice injected with WT (*n* = 7, 4/7 tumors were <0.5 cm^3^ at the endpoint) versus Tslpr^KO^ (*n* = 8, 0/8 tumors were <0.5 cm^3^ at the endpoint) CD4^+^ T cells. The duration of treatment is indicated by purple bars on the graphs. **(D)** Schematic diagram of the experimental paradigm used to elucidate the efficacy of calcipotriol treatment for the suppression of established breast tumor. Note that the calcipotriol treatment was started when implanted tumors became palpable (∼5 mm in diameter) and was repeated every 3 d for 4 wk. Transferred WT CD4^+^ T cells express CD45.1. **(E)** Spider plot of PyMtOva^tg^ Tslpr^KO^ primary tumor growth in Tslpr^KO^ mice injected with WT (*n* = 6, 3/6 tumors were <0.5 cm^3^ at the endpoint) versus Tslpr^KO^ (*n* = 6, 0/6 tumors were <0.5 cm^3^ at the endpoint) CD4^+^ T cells and treated with calcipotriol (two-way ANOVA). The duration of calcipotriol treatment is indicated by purple bar on the graph. **(F)** Survival rate of Tslpr^KO^ mice injected with WT (*n* = 6) versus Tslpr^KO^ (*n* = 6) CD4^+^ T cells followed by PyMtOva^tg^ Tslpr^KO^ primary tumor implantation and calcipotriol treatment (log-rank test). The duration of calcipotriol treatment is indicated by purple bar on the graph. **(G)** Distribution of histological grades of PyMtOva^tg^ Tslpr^KO^ primary tumors developed in Tslpr^KO^ mice injected with WT (*n* = 6) versus Tslpr^KO^ (*n* = 6) CD4^+^ T cells and treated with calcipotriol (Fisher’s exact test). **(H)** Representative images of H&E-stained PyMtOva^tg^ Tslpr^KO^ breast tumors at low (scale bar: 1 mm) and high (scale bar: 100 μm) magnifications from Tslpr^KO^ mouse injected with WT CD4^+^ T cells and treated with calcipotriol, Tslpr^KO^ mouse injected with Tslpr^KO^ CD4^+^ T cell and treated with calcipotriol, and the donor tumor from PyMtOva^tg^ Tslpr^KO^ mouse (scale bar: 100 μm). **(I)** Representative flow plots of transcriptional factor expression in CD4^+^ T cells isolated from PyMtOva^tg^ Tslpr^KO^ breast tumor from Tslpr^KO^ mice injected with CD45.1^+^ WT versus TSLPR^KO^ CD4^+^ T cells and treated with calcipotriol. Numbers on the plots represent the percentage of cells within each gate. **(J and K)** Percentage GATA3^+^ Th2 cells (J) and Foxp3^+^ regulatory T cells (Tregs; K) among CD45.1^+^ WT CD4^+^ T cells (test, *n* = 7) versus Tslpr^KO^ CD4^+^ T cells (control, *n* = 6) isolated from PyMtOva^tg^ Tslpr^KO^ breast tumors and their draining lymph nodes in Tslpr^KO^ mice treated with calcipotriol (Mann–Whitney *U* test). Bar graph shows mean + SD. Each of the tumors in the studies is from a separate mouse. All experimental data verified in at least two independent experiments. *, P < 0.05; **, P < 0.01; ***, P < 0.0001.

To determine whether transient induction of Th2 immunity by TSLP is sufficient for a durable response against breast carcinogenesis, we induced systemic TSLP using a Food and Drug Administration–approved topical medication, calcipotriol, which is known to stimulate the production of TSLP by epidermal keratinocytes in mice (Fig. S5 A; Li et al., 2006). Tslpr^KO^ mice received WT (test) or Tslpr^KO^ (control) CD4^+^ T cells followed by PyMtOva^tg^ Tslpr^KO^ tumor implantation. Starting 2 d after implantation, all animals were treated with topical calcipotriol every 3 d for 4 wk (Fig. 7 A). At the end of the topical treatment, mice continued to be monitored for an additional month. Calcipotriol treatment markedly suppressed PyMtOva^tg^ Tslpr^KO^ tumor growth in Tslpr^KO^ mice that received WT CD4^+^ T cells (P = 0.0029; Fig. 7 B). Tumor suppression was maintained for >30 d after the treatment was stopped, corresponding to the long-term survival of the majority of mice in the test group (P = 0.0173; Fig. 7 C). Importantly, the persistent tumor suppression was mediated by the reversion of the breast tumors into low-grade fibrocystic structures surrounded by significant CD4^+^ T cells in the test group compared with high-grade tumors in the controls (P = 0.0119; Fig. S5, B and C).

Next, we tested whether calcipotriol treatment started at a later time point during tumor development can revert high-grade established tumors into low-grade tumors. A 4-wk treatment with calcipotriol starting when the tumors became palpable (∼5 mm in diameter) led to significant suppression of PyMtOva^tg^ Tslpr^KO^ breast cancer growth in Tslpr^KO^ recipient mice that received WT compared with Tslpr^KO^ CD4^+^ T cell transfer (P = 0.0067; Fig. 7, D and E). This tumor suppression was maintained for >30 d after the treatment was stopped, corresponding to the long-term survival of 50% of the mice in the test group (P = 0.0293; Fig. 7 F). The persistent tumor suppression was mediated by the reversion of the breast tumors into low-grade fibrocystic structures accompanied by the accumulation of CD45.1^+^ WT Th2 cells in the test compared with control tumors (P = 0.037; Fig. 7, G–K). No inflammation affected the adjacent normal mammary glands, skin, or lung of the calcipotriol-treated animals (Fig. S5, D and E). These findings demonstrate that a short course of TSLP induction is safe and effective in establishing long-lasting Th2 immunity against breast cancer.

### TSLP expression by mammary epithelial cells protects against breast carcinogenesis

**Figure 8.**
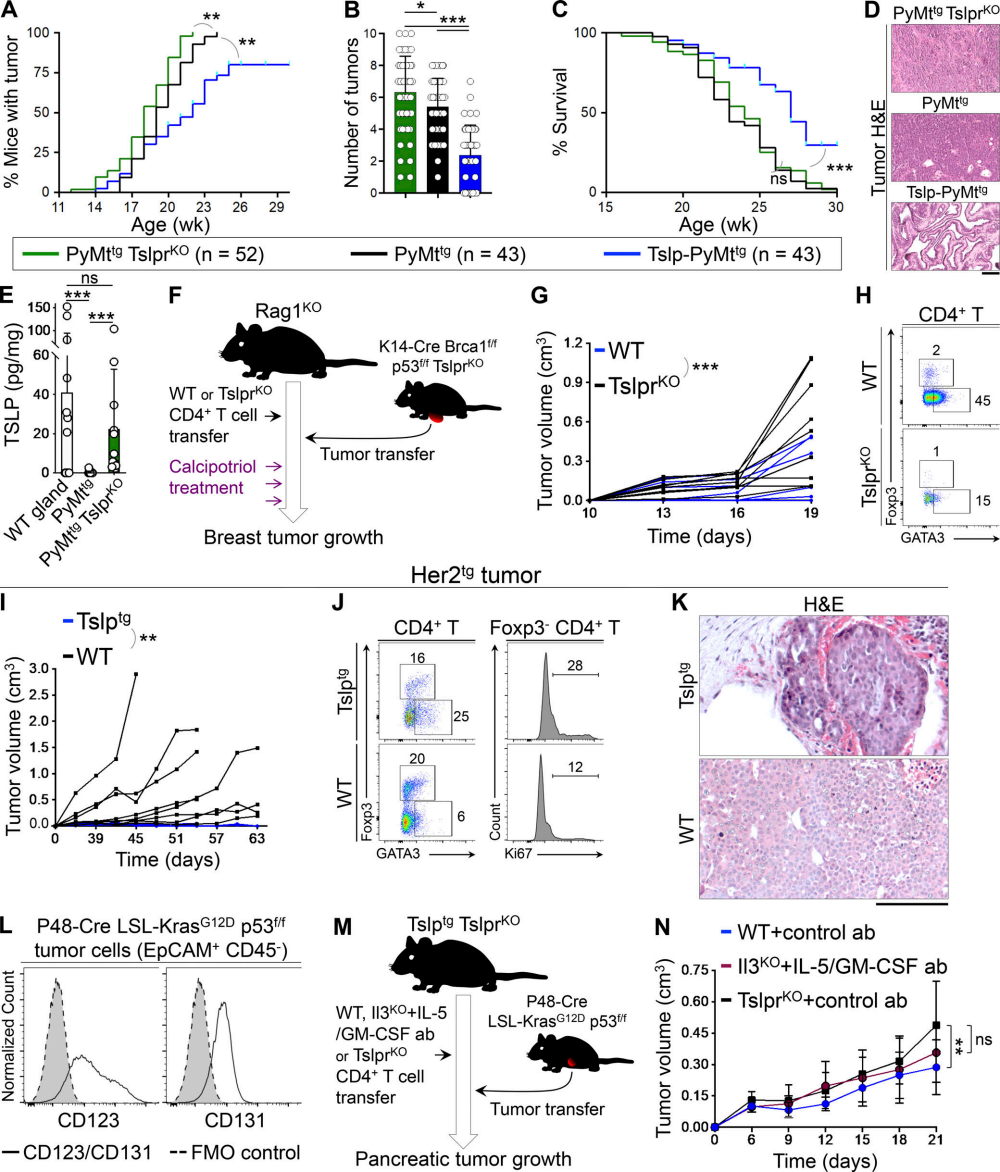
**Endogenous and induced TSLP activate Th2 immunity against primary cancer development. (A–C)** PyMt^tg^ Tslpr^KO^, PyMt^tg^, and Tslp-PyMt^tg^ mice on C57BL/6 background are compared for time to tumor onset (log-rank test; A), number of tumors per mouse (unpaired *t* test; B), and percentage survival (log-rank test; C). Bar graph shows mean + SD. **(D)** Representative images of H&E-stained PyMt^tg^ Tslpr^KO^, PyMt^tg^, and Tslp-PyMt^tg^. Note the glandular (i.e., differentiated) nature of the breast tumor in Tslp-PyMt^tg^ group (scale bar: 100 μm). **(E)** TSLP protein levels per mg of total tissue protein measured with ELISA in WT mammary glands (*n* = 12), PyMt^tg^ breast tumor (*n* = 12), and Tslp-PyMt^tg^ breast tumor (*n* = 12, Mann–Whitney *U* test). Bar graph shows mean + SD. **(F)** Schematic diagram of the experimental paradigm used to examine the impact of topical calcipotriol-induced CD4^+^ T cell immunity against *Brca1*-defcient primary breast tumors. **(G)** Spider plot showing K14-Cre Brca1^f/f^ p53f^/f^ Tslpr^KO^ breast tumor growth in Rag1^KO^ mice injected with WT (test, *n* = 9, 9/9 tumors were <0.5 cm^3^ at the endpoint) versus Tslpr^KO^ (control, *n* = 9, 4/9 tumors were <0.5 cm^3^ at the endpoint) CD4^+^ T cells (two-way ANOVA). **(H)** Representative flow plots of transcriptional factor expression in the tumor-infiltrating CD4^+^ T cells isolated from Rag1^KO^ mice injected with WT versus TSLPR^KO^ CD4^+^ T cells. Numbers on the plots represent the percentage cells within each gate. **(I)** Spider plot of Her2^tg^ primary breast tumor growth in Tslp^tg^ (*n* = 6, 6/6 tumors were <0.5 cm^3^) versus WT (*n* = 8, 3/8 tumors were <0.5 cm^3^ at the endpoint) mice (two-way ANOVA). **(J)** Representative flow plots of transcription factor expression in the Her2^tg^ tumor-infiltrating CD4^+^ T cells isolated from Tslp^tg^ and WT mice. Histograms showing percentage Ki67^+^ Foxp3^−^ CD4^+^ T effector cells isolated from Tslp^tg^ and WT mice. Numbers on the plots represent the percentage of cells within each gate. **(K)** Representative images of H&E-stained Her2^tg^ breast tumors developed in Tslp^tg^ versus WT mice (scale bar: 100 μm). **(L)** Histogram showing expression levels of mouse IL-3Rα (CD123) and the common β chain receptor (CD131) on the surface of EpCAM^+^ CD45^−^ P48-Cre LSL-Kras^G12D^ p53^f/f^ pancreatic tumor cells. Gray histograms with dashed outline show FMO control. **(M)** Schematic diagram of the experimental paradigm used to elucidate the effect of WT CD4^+^ T cells and the impact of IL-3/IL-5/GM-CSF blockade on pancreatic tumor growth in response to TSLP induction. Note that the transferred WT CD4^+^ T cells express CD45.1. **(N)** Graph of P48-Cre LSL-Kras^G12D^ p53^f/f^ primary tumor growth in Tslp^tg^ Tslpr^KO^ mice injected with WT (*n* = 8), Il3^KO^ (*n* = 7), or Tslpr^KO^ (*n* = 8) CD4^+^ T cells. Mice that received Il3^KO^ CD4^+^ T cells were treated with IL-5 and GM-CSF blocking antibodies, while mice that received WT and Tslpr^KO^ CD4^+^ T cells were treated with control antibody (two-way ANOVA). Each of the tumors in the studies is from a separate mouse. All experimental data verified in at least two independent experiments. *, P < 0.05; **, P < 0.01; ***, P < 0.0001.

TSLP was expressed in the normal breast gland and was lost during early breast cancer development (Fig. S5 F). To investigate the role of baseline TSLP in breast carcinogenesis, we examined spontaneous breast tumor development in PyMt^tg^ Tslpr^KO^ mice compared with PyMt^tg^ and Tslp-PyMt^tg^ animals on the C57BL/6 background. Loss of baseline TSLP signaling in PyMt^tg^ Tslpr^KO^ mice led to significantly earlier breast tumor onset (P = 0.0065; Fig. 8 A) and increased tumor numbers per animal compared with PyMt^tg^ mice (P = 0.0353; Fig 8 B). However, the survival and terminal tumor grades were not affected in PyMt^tg^ Tslpr^KO^ compared with PyMt^tg^ mice (Fig. 8, C and D). As expected, the induction of TSLP in Tslp-PyMt^tg^ mice resulted in markedly delayed tumor onset (P = 0.0003; Fig. 8 A), fewer breast tumors per animal (P < 0.0001; Fig. 8 B), and extended survival associated with low-grade breast tumors compared with PyMt^tg^ and PyMt^tg^ Tslpr^KO^ mice (P < 0.0001; Fig. 8, C and D). Consistent with a protective role for baseline TSLP expression by mammary epithelial cells against early phases of breast cancer development, we found a significant reduction in TSLP protein levels in terminal PyMt^tg^ breast tumors compared with WT mammary glands (P < 0.0001; Fig. 8 E). Interestingly, TSLP levels in terminal PyMt^tg^ Tslpr^KO^ tumors were restored back to WT mammary gland levels, suggesting that TSLP loss in breast cancer is due to negative selection against this cytokine to escape TSLP-induced Th2 cell immunity during the early malignant transformation (Fig. 8 E).

To investigate the role of TSLP in the early stages of tumor development in other breast cancer models, we developed a T cell/primary tumor transfer system in which Rag1^KO^ mice received WT (test) or Tslpr^KO^ (control) CD4^+^ T cells followed by primary breast tumor implantation from K14-Cre Brca1^f/f^ p53^f/f^ Tslpr^KO^ mice into their abdominal mammary fat pad. 2 d later, all animals were treated with topical calcipotriol every 3 d (Figs. 8 F and S5 G). Rag1^KO^ mice that received WT CD4^+^ T cells were protected from Brca1-deficient breast tumor growth accompanied by the development of low-grade tumors compared with the Rag1^KO +^ Tslpr^KO^ CD4^+^ T cell control group (P = 0.0001; Figs. 8 G and S5 H). K14-Cre Brca1^f/f^ p53^f/f^ Tslpr^KO^ tumor suppression in the test group was associated with the infiltration of GATA3^+^ Th2 cells in the tumors (Fig. 8 H and Fig. S5, I and J). We found similar tumor suppression by TSLP-stimulated Th2 cells against *MMTV-HER-2/neu*^*tg*^ (Her2^tg^; Guy et al., 1992) breast tumors, which was associated with low-grade tumors lacking growth potential (Fig. 8, I–K).

To extend our mechanistic discoveries from breast cancer to other cancer models, we investigated the impact of TSLP-activated CD4^+^ T cells and their downstream effector cytokines (IL-3, IL-5, and GM-CSF) on a well-established pancreatic cancer model (P48-Cre LSL-Kras^G12D^ p53^f/f^; Hingorani et al., 2003). Common β chain (CD131) and IL-3Rα (CD123) were expressed on the surface of P48-Cre LSL-Kras^G12D^ p53^f/f^ pancreatic tumor cells (Fig. 8 L). Importantly, TSLP-activated WT CD4^+^ Th2 cells suppressed P48-Cre LSL-Kras^G12D^ p53^f/f^ pancreatic tumor growth compared with Tslpr^KO^ CD4^+^ T cells in Tslp^tg^ Tslpr^KO^ recipient mice (Fig. 8, M and N; and Fig. S5, K and L). This protection was reversed in Tslp^tg^ Tslpr^KO^ mice that received Il3^KO^ CD4^+^ T cells plus IL-5– and GM-CSF–blocking antibodies (Fig. 8, M and N; and Fig. S5, K and L).

### TSLP is expressed by human mammary epithelial cells and is lost during human breast cancer development

**Figure 9.**
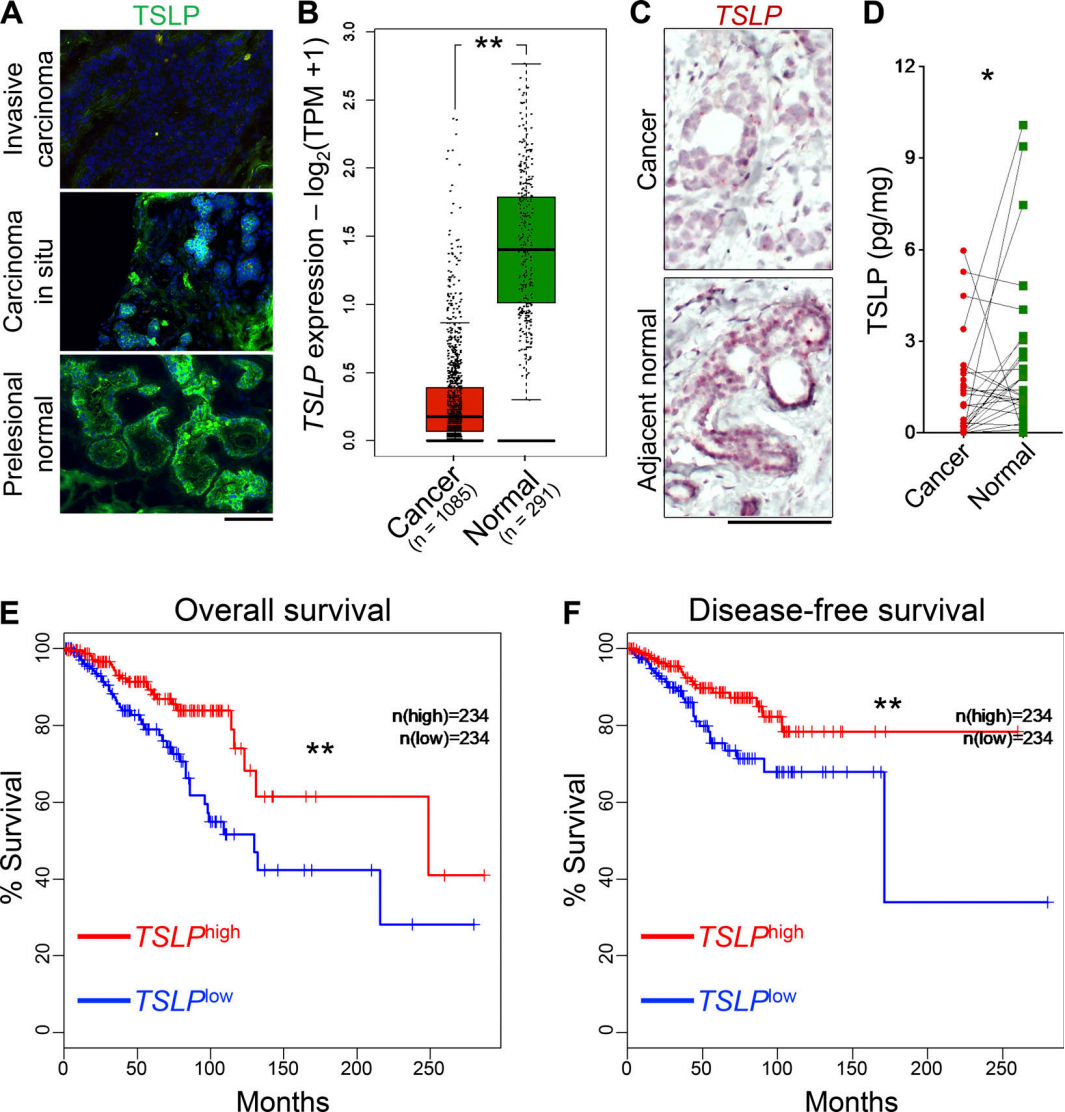
**Loss of TSLP expression in human breast cancer cells is associated with cancer progression and worse survival outcomes. (A)** Immunofluorescence staining for TSLP in human breast tissue at three stages of cancer development. Note the complete loss of TSLP in invasive carcinoma (scale bar: 100 μm). **(B)** Box plot of *TSLP* expression in normal mammary glands versus breast cancers across TCGA/GTEx datasets (one-way ANOVA, Gene Expression Profiling Interactive Analysis database). TPM, transcript count per million. **(C)** *TSLP* RNA in situ hybridization showing *TSLP* expression in the breast cancer (upper panel) compared with its matched adjacent normal tissue (lower panel). Note that the normal mammary epithelial cells are the dominant source of *TSLP* transcripts in the breast (scale bar: 100 μm). **(D)** Normalized TSLP protein levels measured with ELISA in the paired samples of breast cancer and adjacent normal breast tissue from 28 patients with primary breast cancer (Wilcoxon test). **(E)** Overall survival of patients with basal-like, luminal A, luminal B, and HER2 breast cancers divided based on high (top 30%, *n* = 234) and low (bottom 30%, *n* = 234) tumor *TSLP* expression levels in TCGA (log-rank test, Gene Expression Profiling Interactive Analysis database). **(F)** Disease-free survival of patients with basal-like, luminal A, luminal B, and HER2 breast cancers divided based on high (top 30%, *n* = 234) and low (bottom 30%, *n* = 234) tumor *TSLP* expression levels in TCGA (log-rank test, Gene Expression Profiling Interactive Analysis database). All experimental data verified in at least two independent experiments. *, P < 0.05; **, P < 0.01; ***, P < 0.0001.

Finally, we examined TSLP expression in normal mammary glands and breast cancers in humans. Consistent with our findings in mice, TSLP expression by mammary epithelial cells was lost during the early phases of breast cancer promotion (Fig. 9 A). *TSLP* loss in breast cancers compared with normal glands was validated across a large collection of breast tissue samples represented in the Cancer Genome Atlas (TCGA) and the Genotype-Tissue Expression (GTEx) databases (Fig. 9 B). The loss of *TSLP* expression was noted across all breast cancer subtypes represented in TCGA (Fig. S5 M). Using *TSLP* RNA in situ hybridization assays, we documented the loss of epithelial *TSLP* expression in the matched samples of primary breast tumors compared with their adjacent normal mammary glands (Fig. 9 C). Consistent with this finding, TSLP protein levels were significantly reduced in the human breast cancers compared with their adjacent normal mammary tissues (P = 0.0385; Fig. 9 D). Although TSLP levels are overall reduced in breast cancer, high *TSLP* expression significantly correlated with increased overall and disease-free survival among breast cancer patients represented in TCGA (Fig. 9, E and F). In addition, we found positive correlations between *TSLP* expression and *CSF2*/*IL3*/*IL5* and differentiation genes expression in human breast cancer (Fig. S5, N and O). Collectively, these data reveal a negative selection against TSLP expression during early breast cancer development in humans, which can be reversed by TSLP induction to prevent and treat breast cancer.

## Discussion

Our findings demonstrate that TSLP-stimulated CD4^+^ T cell immunity blocks breast cancer promotion by engulfing primary breast tumors and transforming them into low-grade, fibrocystic structures, with no metastatic potential. We show that CD4^+^ T cells are sufficient to establish this tumor-suppressive phenotype, which depends on Th2 polarization and persists in the absence of CD8^+^ T or B cells. Instead of cytotoxicity, CD4^+^ Th2 cells directly block breast carcinogenesis by inducing the terminal differentiation of breast cancer cells. This novel effector mechanism is mediated by tumor-infiltrating Th2 cells releasing IL-3, IL-5, and GM-CSF, which bind to a shared receptor on breast tumor cells. We find that baseline TSLP, which is expressed by mammary epithelial cells and actively lost in breast cancers of mice and humans, has a similar tumor protective effect. Finally, we show that high *TSLP* expression in the human breast cancer is associated with improved survival. Together, these outcomes establish the antitumor function of Th2 cells in early cancer development, which can be leveraged for cancer immunoprevention and treatment.

The discovery of Th2 cells as direct mediators of antitumor immunity in early breast carcinogenesis provides fundamental insights into the importance of Th2 cells in maintaining tissue homeostasis. In contexts in which Th2 cells have been associated with cancer progression, the main protumorigenic effector cell types are B cells and innate immune cells, which are present in type 2 inflammation (Johansson et al., 2008). In such tumor-promoting immune environments, Th2 cells are found to have a regulatory profile associated with high levels of IL-10 and TGF-β (Johansson et al., 2008). In contrast, we find Th2 cells with high expression of IL-13, IL-3, IL-5, and GM-CSF (i.e., inflammatory Th2 cells; Ito et al., 2005) that can effectively protect the breast gland against oncogene-driven malignant transformation. Although Th2 cell contribution to tumor suppression has been previously associated with their ability to activate eosinophils as the cytotoxic effector cells (Hung et al., 1998; Tepper et al., 1992), we find Th2 cells directly block cancer development in the breast by terminally differentiating the tumor cells to form gland-like structures. Th2 cell–induced terminal differentiation is driven by IL-3/IL-5/GM-CSF binding to receptors with a common β chain on breast cancer cells, which results in the activation of the signaling transducer and activator of transcription 5 (STAT5) pathway (Broughton et al., 2012). STAT5 signaling is an essential pathway in maintaining mammary gland development (Haricharan and Li, 2014), and its activation in human breast cancer is associated with higher differentiation status and favorable clinical outcomes (Peck et al., 2011; Yamashita et al., 2006). Therefore, Th2 cells invoke a fundamental homeostatic mechanism to protect the mutated mammary epithelial cells from cancer. In addition to its essential role in polarization of TSLP-stimulated CD4^+^ T cells, we find that baseline IL-4/13 signaling, which is also implicated in normal mammary gland development (Haricharan and Li, 2014), can protect against breast cancer development. These findings warrant further investigation, as they raise concern about breast cancer risk in patients who are receiving IL-4rα blocking antibodies for the treatment of allergic diseases (Beck et al., 2014; Wenzel et al., 2013).

CD4^+^ T cell activation for cancer immunoprevention and therapy has several distinct advantages compared with conventional immunotherapies directed at CD8^+^ T cells. CD4^+^ T cells are upstream activators of adaptive immunity, and their direct activation and targeting of the tumor antigens can initiate a robust antitumor immune response in cold tumors, which include early epithelial cancer and precancerous lesions (Kennedy and Celis, 2008). As shown in the context of autoimmunity (Rogers and Unanue, 1993), CD4^+^ T cells are essential to initiate the immune responses that are subsequently dominated by CD8^+^ T cells during their later stages. Thus, CD4^+^ T cell activation in established cancers can transform “cold” tumors into “hot” immunogenic tumors that are responsive to conventional immunotherapeutics. This concept is supported by findings on the essential role of MHC class II–restricted neoantigens in shaping tumor immunity and mediating responsiveness to immunotherapy (Alspach et al., 2019). Determining whether the activation of inflammatory Th2 cells augments the efficacy of current immunotherapeutic approaches against advanced cancers requires future investigations.

This work establishes that systemic TSLP induction activates a tumor antigen–specific Th2 cell immunity, which has a dominant role in suppressing the early stages of breast carcinogenesis. Previously, research on the effect of TSLP in breast cancer using breast cancer cell lines showed a protumorigenic function for TSLP expressed in the tumor microenvironment (Demehri et al., 2016; Olkhanud et al., 2011; Pedroza-Gonzalez et al., 2011). In a more recent report, this progrowth function of TSLP was linked to a tumor-myeloid cell axis that is independent of T cell responses (Kuan and Ziegler, 2018). Genetic and chemical induction of systemic TSLP released from skin keratinocytes leads primarily to the activation of Th2 cells that specifically target breast cancer cells, block breast cancer promotion, and revert high-grade tumors into low-grade, fibrocystic structures in the absence of any inflammation, affecting the normal breast glands in close proximity to the tumor foci. The efficacy of transient and topical TSLP induction in delivering a lasting tumor-specific immunity in the breast highlights the potential for the use of TSLP inducers such as calcipotriol as safe and accessible agents for breast cancer immunoprevention.

Terminal differentiation as the mode of immunity imposed by TSLP-stimulated Th2 cells against malignant cells in the breast has major implications for preventing breast cancer progression and recurrence in patients. The histological grade of a primary breast cancer is closely associated with its clinical outcomes, including cancer recurrence, metastasis, and disease-free survival (Bombonati and Sgroi, 2011; Pan et al., 2017). A high tumor grade is an independent variable that is significantly correlated with earlier breast cancer recurrence (Fitzpatrick et al., 2014). In addition, the histological grade of primary ER-positive/HER2-negative breast tumors has been shown to be an independent predictor of relapse-free survival (hazard ratio = 2.13, 95% confidence interval, 1.79–2.53; P < 0.0001; Rakha et al., 2008; Rakha et al., 2010). Therefore, inducing Th2 cell immunity in the breast can be readily translated into improved clinical outcomes for early and late breast cancer patients by preventing the development of high-grade tumors and reverting already formed high-grade cancers into low-grade fibrocystic structures with reduced recurrence or metastatic potential. However, it is essential to examine the impact of TSLP/Th2 cell axis in metastatic breast cancer models to fully capture its therapeutic potential. In addition, it is important to determine whether other cellular changes such as senescence induction contribute to antitumor Th2 cell immunity in the breast. Furthermore, the specific role of IL-3 and IL-5 versus GM-CSF to breast cancer suppression and the contributions of antigen-nonspecific T cell response and other immune cell types including eosinophils, macrophages, and CD8^+^ T and NK cells to Th2 cell immunity warrant further investigation. We aim to address these current limitations in our future research.

In summary, our findings establish a previously unrecognized mode of immunity against early malignant transformation, which is mediated directly by Th2 cells. TSLP, as an epithelium-derived alarmin, induces a robust and tumor-specific CD4^+^ T cell immunity against breast cancer. This highlights the importance of the immune activating signals released by epithelial cells as early initiators of antitumor immunity, which can be therapeutically leveraged for cancer immunoprevention and treatment.

## Materials and methods

### Study approval

Animal studies were approved by Massachusetts General Hospital (MGH) Institutional Animal Care and Use Committee. Analysis of de-identified human tissues was reviewed and approved by MGH Institutional Review Board.

### Mice

All mice were housed under specific pathogen–free conditions with 12-h light-dark cycle and given water and food ad libitum, in the animal facility at MGH in compliance with animal care and all other relevant regulations. *MMTV-PyMT*^*tg*^ (PyMt^tg^, a gift of Dr. David DeNardo, Washington University in St. Louis, St. Louis, MO), *MMTV-PyMT-mCherry-Ova*^*tg*^ (PyMTChOva^tg^, a gift of Dr. Matthew Krummel, University of California San Francisco, San Francisco, CA), *K14-TSLP*^*tg*^ (Tslp^tg^, a gift of Dr. Andrew Farr, University of Washington, Seattle, WA), and *Tslpr*^*−/−*^ (Tslpr^KO^, a gift of Dr. Warren Leonard, National Institutes of Health, Bethesda, MD) were obtained from other academic laboratories. *MMTV-HER-2/neu*^*tg*^ (Her2^tg^, IMSR_JAX:002376), *Rag1*^*tm1Mom*^*/J* (Rag1^KO^, IMSR_JAX:002216), *Brca1*^*tm1Aash*^*/J* (Brca1^f/f^, IMSR_JAX:017835), *Il3*^*tm1Tyb*^*/J* (Il3^KO^, IMSR_JAX:026277), *Il4*^*tm1Nnt*^*/J* (Il4^KO^), *Ifng*^*tm1Ts*^*/J* (Ifng^KO^, IMSR_JAX:002287), *Tnf*^*tm1Gkl*^*/J* (Tnf^KO^, IMSR_JAX:005540), *Rag2*^*tm1Fwa*^ *Tg(TcraTcrb)425Cbn* (OTII Rag2^KO^, IMSR_JAX:004194), *Il4ra*^*tm1Sz*^*/J* (Il4r^KO^, IMSR_JAX:002518), *Tg(KRT14-cre)1Amc/J* (K14-Cre, IMSR_JAX:004782), *Trp53*^*tm1Brn*^*/J* (p53^f/f^, IMSR_JAX:008462), *Ptprc*^*a*^ *Pepc*^*b*^*/BoyJ* (CD45.1^+^ WT, IMSR_JAX:002014), Csf2rb2^*tm1Cgb*^ Csf2rb2^*tm1Clsc*^*/J* (Bc/BIL3^KO^, IMSR_JAX:005963), Ptf1a^*tm1(cre)Hnak*^*/RschJ* (P48-Cre, IMSR_JAX:023329), and Kras^*tm14Tyj*^*/J* (LSL-Kras^G12D^, IMSR_JAX:008179) mice were purchased from certified vendors and bred and maintained in our facility to obtain mice used in the studies. PyMt^tg^ and Tslp-PyMt^tg^ mice were maintained on the BALB/c and C57BL/6 backgrounds. PyMt^tg^ and Tslp-PyMt^tg^ mice with Rag1^KO^ or Il4r^KO^ genotypes were maintained on the BALB/c background. PyMtOva^tg^, Tslpr^KO^, Il3^KO^, Il4^KO^, Ifng^KO^, Tnf^KO^, OTII Rag2^KO^, K14-Cre Brca1^f/f^ p53^f/f^, Rag1^KO^, Csf2rb^KO^ Csf2rb2^KO^, and CD45.1^+^ WT mice were maintained on the C57BL/6 background. Her2^tg^ mice were kept on FVB (Friend leukemia virus B) background. Age-matched female mice were used in all the breast cancer studies.

### Human tissue analysis

Deidentified human breast cancer and healthy breast tissue samples used in the study were obtained from the pathology department at MGH. Frozen tissue sections (9 μm) in optimal cutting temperature (OCT) compound were used for TSLP immunofluorescence staining (Table S1). 5 μm formalin-fixed paraffin-embedded tissue was used for RNA in situ hybridization. Proteins for TSLP ELISA were extracted from fresh tissues and quantified using Pierce BCA Protein Assay Kit (Thermo Fisher Scientific), according to manufacturers’ instructions.

### Spontaneous breast cancer studies

Tumor onset and tumor growth were monitored weekly. Tumor volume was calculated in cm^3^ using the formula (length [mm] × width [mm]^2^)/2,000, as previously described (Faustino-Rocha et al., 2013). Animals were harvested once their tumors reached 2 cm in diameter or they showed any sign of distress or weight loss. Blood was collected retro-orbitally using heparinized capillaries, and plasma was isolated by centrifugation of the blood at 2,000 *g* for 15 min and stored at −80°C for future analysis. Skin, lungs, and breast tumor were collected in 4% paraformaldehyde (Sigma-Aldrich) for histological analysis. Tumor and tumor-draining lymph nodes were harvested for flow cytometric analysis and for CD4^+^ T cell sorting. Breast tumors were also collected for RNA and protein isolation. Breast tumors from PyMt Tslpr^KO^, PyMtOva Tslpr^KO^, PyMt Il4r^KO^, PyMt^tg^, K14-Cre Brca1^f/f^ p53^f/f^ Tslpr^KO^, PyMtOva Csf2rb^KO^ Csf2rb2^KO^, and Her2^tg^ mice were collected, minced, and viably frozen in liquid nitrogen for primary tumor implantation experiments.

### CD4^+^ T cell sorting and supernatant collection

Sorted CD4^+^ T cells were stimulated and rested ex vivo, and supernatants derived from these cultures were used for coculture experiments and for protein analysis. In details, tumor and tumor-draining lymph nodes collected from Tslp-PyMt^tg^, PyMt^tg^ Tslpr^KO^, Il3^KO^, and WT mice were processed into a single-cell suspension and stained with anti-CD3ε, anti-CD4, anti-CD8α, anti-CD19, anti-NKp46, anti-NK1.1, and anti-CD49b (Table S1). Il3^KO^ and WT mice were first treated with topical calcipotriol (10 nmol in 100% ethanol on the back skin; Sigma-Aldrich) and subcutaneous OVA (50 μg in PBS; Sigma-Aldrich) every 3 d for 3 wk before CD4^+^ T cell isolation from the skin-draining lymph nodes. CD4^+^ T cells were sorted on a BD FACSAria (BD Biosciences) or SONY SH800 sorter (Sony Biotechnologies), and plated at the concentration of 0.5 × 10^6^ cells/ml in anti-CD3 (Table S1)–coated plates in RPMI 1640 (Gibco), supplemented with 10% FBS (Corning), 1% penicillin/streptavidin/glutamine (Thermo Fisher Scientific), 22 μM 2-mercaptoethanol (Gibco), 50 U/ml IL-2 (BioLegend), 20 ng/ml TSLP (Thermo Fisher Scientific), and 2 μg/ml anti-CD28 (Table S1). After 72 h, T cell culture supernatants were harvested, filtered through a 22-μm filter, and stored at −20°C until use. Cells were plated again at 0.5 × 10^6^ cells/ml in 12-well plates in the same medium without anti-CD3/CD28 and cultured for 48 h. The stimulation-resting cycle was repeated for a total of three stimulations, and supernatants were collected at the end of each stimulation.

### Secretome analysis

For secretome analysis, cells were treated as previously described (Eichelbaum et al., 2012). Briefly, at the end of the third stimulation, cells were rested for 24 h, and then plated in RPMI 1640 without L-arginine, L-lysine, L-leucine, and L-methionine (Athena Enzyme Systems), supplemented with 10% FBS (Gibco), 0.1 mM azidohomoalanine (AnaSpec), 84 μg/ml L-arginine, and 146 μg/ml L-lysine (Athena Enzyme Systems) for 18 h. Supernatants were collected, filtered, and stored at −80°C until use. Total protein was quantified using a Pierce BCA Protein Assay Kit (Thermo Fisher Scientific). Newly translated and secreted proteins were isolated from supernatants using the Click-iT Protein Enrichment Kit (Thermo Fisher Scientific) following manufacturer instructions. Immobilized proteins were reduced, alkylated, and digested with LysC and trypsin as previously described (Lapek et al., 2017). 15 μg of the resulting peptides per sample were subsequently labeled using TMT-10plex reagents (Thermo Fisher Scientific; Lapek et al., 2017). Labeled samples were pooled, desalted, and analyzed in a 3-h reversed-phase LC-MS2/SPS-MS3 run on an Orbitrap Lumos mass spectrometer (McAlister et al., 2014; Ting et al., 2011). MS2 spectra were assigned peptide sequences using a SEQUEST-based in-house-built proteomics data analysis platform and the Uniprot database of human protein sequences (downloaded February 2014; Huttlin et al., 2010). MS2 search filtering an validation was performed using the target-decoy database-based search strategy to achieve false discovery rates of <1% for peptide and protein identification (Elias and Gygi, 2007). Peptide and protein quantification were performed based on MS3 data (Lapek et al., 2017).

### Adoptive T cell transfer

Naive CD4^+^ and CD8^+^ T cells from spleens of donor mice were used for adoptive T cell transfers. A single-cell suspension was obtained from spleens, and cells were stained with anti-CD3ε, anti-CD4, anti-CD8α, anti-CD19, anti-NKp46, and anti-CD49b (Table S1). CD3^+^CD4^+^CD19^−^NKp46^−^CD49b^−^ and CD3^+^CD8^+^CD19^−^NKp46^−^CD49b^−^ cells were sorted using a BD FACSAria II sorter (BD Biosciences). Sorted CD4^+^ T cells were injected retro-orbitally into 4-wk-old PyMt Rag^KO^ and Tslp-PyMt Rag^KO^ mice, and sorted CD8^+^ T cells were injected retro-orbitally into 4-wk-old Tslp-PyMt Rag^KO^ mice. Mice were monitored weekly for tumor onset, tumor numbers and tumor volume.

### Adoptive T cell transfer and tumor implantation experiments

Recipient Tslp^tg^ Tslpr^KO^ or Tslpr^KO^ mice were sublethally irradiated with 450 cGy on day 0 using a ^137^Cs irradiator. 24 h later, spleen was isolated from CD45.1^+^ WT, Tslpr^KO^, Il3^KO^, Il4^KO^, Ifng^KO^, Tnfa^KO^, or OTII Rag2^KO^ donor mice and processed to obtain a single-cell suspension. CD4^+^ T cells were enriched using the MojoSort Mouse CD4 T Cell Isolation Kit (BioLegend) and then stained with anti-CD3ε, anti-CD4, anti-CD8, anti-CD45.1 (for CD45.1^+^ WT mice), anti-CD19, anti-CD49b, and anti-NK1.1 (Table S1) monoclonal antibodies for cell sorting. CD3^+^CD4^+^ T cells were sorted on a BD FacsARIA (BD Biosciences) or SONY SH800 sorter (Sony Biotechnologies). Sorted CD3^+^CD4^+^ T cells were transferred into the recipient mice by i.v. injection under isoflurane anesthesia. 1 d after T cell transfer, a tumor single-cell suspension was prepared by thawing frozen primary tumors and incubating them in Collagenase IV (Worthington Biochemical Corp.) for 1 h in a 37°C shaker. Digested tumors were then filtered through a 70-μm cell strainer, washed, counted, and resuspended in PBS for injection. A single-cell suspension of 1 × 10^6^ PyMtOva^tg^ Tslpr^KO^, PyMt^tg^ Tslpr^KO^, or PyMtOva^tg^ Csf2rb^KO^ Csf2rb2^KO^ tumor cells mixed 1:1 vol/vol with Matrigel (Corning) were orthotopically injected into the left abdominal mammary fat pad of Tslp^tg^ Tslpr^KO^ or Tslpr^KO^ mice under isoflurane anesthesia. For Brca1 tumor transfer, Rag1^KO^ mice were injected with sorted Tslpr^KO^ or CD45.1^+^ WT CD4^+^ T cells, followed 1 d later by injection of 1 × 10^4^ K14-Cre Brca1^f/f^ p53^f/f^ Tslpr^KO^ primary tumors mixed 1:1 vol/vol with Matrigel (Corning) into the abdominal mammary fat pad of the recipients. For the PyMt IL4r^KO^ tumor transfer, WT or Tslp^tg^ mice were injected in the left abdominal mammary fat pad with a single-cell suspension of 1 × 10^6^ PyMt IL4r^KO^ or PyMt^tg^ tumor cells mixed 1:1 vol/vol with Matrigel (Corning). For IL4r^KO^ T cell transfer experiment, BALB/c Rag1^KO^ mice were injected with sorted BALB/c Il4r^KO^ or WT CD4^+^ T cells, followed 1 d later by injection of 1 × 10^6^ BALB/c PyMt^tg^ primary tumors mixed 1:1 vol/vol with Matrigel (Corning) into the abdominal mammary fat pad of the recipients. For Her2 experiment, recipient Tslp^tg^ and WT F1 (C57BL/6 × FVB) mice were injected with primary 2 × 10^6^ Her2^tg^ tumor cells (FVB). Tumor volume was measured every 3 d, and mice were euthanized when their tumors reached 2 cm in diameter or the mice showed any sign of distress or weight loss. For the pancreatic cancer tumor transfer, Tslp^tg^ Tslpr^KO^ mice were injected in the right flank with 2 × 10^6^ P48-Cre LSL-Kras^G12D^ p53^f/f^ tumor cells mixed 1:1 vol/vol with Matrigel (Corning).

### Depleting antibody treatment

Mice were treated with rat anti-mouse/human IL-5 and rat anti-mouse GM-CSF (Table S1). Antibody treatment was started at the day of T cell transfer and carried out for 7 wk. Anti–IL-5 antibody was injected i.p. every 4 d at a dose of 0.5 mg per mouse, and anti–GM-CSF antibody was injected i.p. every 2 d at a dose of 0.3 mg per mouse. Rat isotype IgG antibodies were injected as control every 2 d alternating a dose of 0.3 and 0.8 mg per mouse.

### Calcipotriol treatment

Tslpr^KO^ mice were treated topically on the tumor with 10 nm calcipotriol (Sigma-Aldrich) dissolved in 100% ethanol every 3 d starting 2 d after tumor transfer or when tumors became palpable (∼5 mm in diameter). After 18 d, the calcipotriol dose was incremented to 20 nm per mouse. For Brca1 tumor transfer and Il4r^KO^ T cell transfer experiments, Rag1^KO^ mice were treated topically on the tumor with 10 nm calcipotriol every 3 d starting 2 d after tumor transfer. After 9 d, the calcipotriol dose was increased to 20 nm. In each experiment, all animals in the test and control groups were treated with the same dose of topical calcipotriol.

### Flow cytometry

Freshly harvested CD45-enriched tumor and lymph node tissues were used for flow analysis. Breast tumors and tumor draining lymph nodes were incubated with collagenase IV (Worthington) and filtered through a 70-μm cell strainer to obtain single-cell suspensions. Tumor-infiltrating leucocytes were isolated by immunomagnetic separation with CD45 MicroBeads on magnetic columns (Miltenyi Biotec). Lymph node and tumor cells were stained with the following monoclonal surface antibodies: anti-CD3ε, anti-CD4, anti-CD8α, anti-CD45, anti-CD19, anti-NKp46, and anti-CD45.1 (Table S1). Next, cells were fixed and permeabilized by True-Nuclear Transcription Factor Buffer Set (BioLegend). After fixation and permeabilization, intracellular stains were performed using the following antibodies: anti-GATA3, anti-Foxp3, and anti-Ki67 (Table S1). Stained cells were assayed using a Fortessa LSRII flow cytometer (BD Bioscience), and the live cell population was gated for analysis on the forward/side scatter plots. GATA3 gates were determined using naive splenic T cells as control. Flow data were analyzed using FlowJo software (TreeStar).

### Histology, immunofluorescence, and immunohistochemistry

Tissue samples were harvested and fixed in 4% paraformaldehyde (Sigma-Aldrich) solution in PBS overnight at 4°C. Samples were processed and embedded in paraffin. Paraffin-embedded tissues were cut at 5 μm and stained for H&E. Slides were stained with toluidine blue (Sigma-Aldrich) to detect mast cells, and with chromotrope 2R (Sigma-Aldrich) to detect eosinophils. For immunofluorescence staining, sections were incubated with anti-mouse or anti-human primary antibodies followed by fluorochrome-conjugated secondary antibodies (Table S1). Sections were counterstained with DAPI nuclear stain (Thermo Fisher Scientific). Slides were scanned using the NanoZoomer s60 digital scanner (Hamamatsu Corp.), and high-resolution images were acquired using a Zeiss Axio Observer Z1 (Zeiss) and analyzed using the Zeiss ZEN Image Processing software. Quantification of cell population was performed with HALO Image Analysis Platform within 200× magnified high-power fields (HPFs; Indica Labs). For immunohistochemistry, slides were immersed in an antigen unmasking solution (Vector Laboratories) at a 1:100 dilution in distilled water. Antigen retrieval was then performed in a Cuisinart high-pressure cooker for ∼20 min. Slides were washed in three 3-min rounds of 1× TBS with 0.025% Triton X-100. For blocking, 1% BSA (Thermo Fisher Scientific) and 5% goat serum (Millipore Sigma) were used for 1 h. Primary antibody (Table S1) was diluted in TBS containing 1% BSA applied overnight. Secondary antibody (Vector Laboratories) was applied after washing the slides as described above for 30 min. Slides were then incubated with 150 μl of reagent A and B mixture from VECTASTAIN Elite ABC universal kit Peroxidase (Vector Laboratories) for 30 min. After washing, the slides were incubated with 150 μl ImmPACT DAB chromogen staining (Vector Laboratories) for 2 min. Counterstaining was performed using hematoxylin. Dehydration was performed by immersing the slides in ethanol, then xylene. Slides were mounted using 2–3 drops of mounting medium.

### RNA and protein isolation

For RNA extraction, breast tumor samples were homogenized in RLT buffer (Qiagen) supplemented with 1% 2-mercaptoethanol (Thermo Fisher Scientific) using a TissueLyser II (Qiagen) at a frequency of 30/s for 5 min. Samples were further lysed by resuspending the homogenized tissue in TRIzol Reagent (Thermo Fisher Scientific) and stored at −80°C until use. Sorted CD4^+^ T cells for RNA sequencing were resuspended in RLT buffer (Qiagen) supplemented with 1% 2-mercaptoethanol (Thermo Fisher Scientific) and stored at −80°C until use. RNA was isolated using the RNeasy Mini Kit (Qiagen), quantified using a NanoDrop ND-1000 spectrophotometer, and stored at −80°C until use.

For protein extraction, breast tumor samples were homogenized in PBS supplemented with 0.1% vol/vol Tween 20 (Sigma-Aldrich) and 4% protease inhibitor (Thermo Fisher Scientific) using a TissueLyser II (Qiagen) at a frequency of 30/s for 5 min. Samples were transferred to new tubes and frozen in liquid nitrogen for 1 min, and then thawed in a 56°C water bath for 3 min. Subsequently, samples were sonicated for 1 min followed by centrifugation at 13,300 rpm for 10 min at 4°C. Supernatant containing protein extract was transferred to a new tube and stored at −80°C for future analysis.

### ELISA

Expression of IL-3, IL-5, IL-13, and GM-CSF cytokines was measured on CD4^+^ T cell supernatants with the Mouse IL-3 Quantikine ELISA Kit (R&D Systems), LEGEND MAX Mouse GM-CSF ELISA Kit, Mouse IL-5 ELISA MAX Deluxe (BioLegend), and Invitrogen IL-13 Mouse ELISA Kit (Thermo Fisher Scientific), following the manufacturers’ instructions. TSLP expression was measured in mouse plasma and breast tumors/mammary glands using the LEGEND MAX Mouse TSLP ELISA Kit (BioLegend). Expression of TSLP in human breast tumor and healthy tissue was measured using the LEGEND MAX Human TSLP ELISA Kit (BioLegend) following manufacturer’s instructions. The same amount of total protein, quantified using Pierce BCA Protein Assay Kit (Thermo Fisher Scientific), was used for the assays. Optical densities were measured on a Synergy Neo2 (BioTek) at 450 nm, and cytokine concentrations were calculated with a five-parameter logistic curve using Gen5 Microplate Reader and Imager Software (BioTek).

### Mammosphere culture

The mouse mammary gland epithelial cell line HC11 (RRID: CVCL_0288) was used for in vitro experiments. HC11 cells were plated in low adherence 48-well plates in RPMI 1640 (Gibco), supplemented with 10% FBS (Corning), 1% penicillin/streptavidin/glutamine (Thermo Fisher Scientific), 22 μM 2-mercaptoethanol (Gibco), and 5 μg/ml insulin (medium) or in CD4^+^ T cell supernatants derived from Tslp-PyMt^tg^ (test), Tslp-PyMt^tg^ Tslpr^KO^, and PyMt^tg^ (control) also supplemented with 5 μg/ml insulin (Sigma-Aldrich). Total protein concentration in the supernatants was quantified using Pierce BCA Protein Assay Kit (Thermo Fisher Scientific) and normalized to have the same amount of protein in each assay. After 5 d, mammospheres were counted under an inverted fluorescence microscope, and pictures of all mammospheres were taken. The size of mammospheres was measured using ImageJ. Mammospheres were collected and centrifuged onto slides using a Cytospin 4 Cytocentrifuge (Thermo Fisher Scientific) at 300 rpm for 3 min. Cells were fixed in methanol at −20°C for 20 min. Slides were permeabilized in PBS supplemented with 0.3% vol/vol Triton X-100 (Thermo Fisher Scientific) for 30 min and blocked with PBS supplemented with 0.1% vol/vol Tween 20 (Sigma-Aldrich), 5% (mass/vol) BSA (Thermo Fisher Scientific), and 10% (vol/vol) goat serum (Sigma-Aldrich) for 1 h. Cells were stained with primary antibodies, mouse anti–E-Cadherin, and Ki67 (Table S1) at 4°C overnight. Slides were incubated with secondary antibodies and incubated with DAPI (Invitrogen) for 10 min. Slides were mounted with Prolong Gold Antifade Reagent (Invitrogen). Pictures were acquired on a Zeiss Axio Observer Z1 (Zeiss) and analyzed with using the Zeiss ZEN Image Processing software.

HC11 cells were plated in low-adherence 48-well plates in RPMI 1640 (Gibco), supplemented with 10% FBS (Corning), 1% penicillin/streptavidin/glutamine (Thermo Fisher Scientific), 22 μM 2-mercaptoethanol (Gibco), and 5 μg/ml insulin (medium) or in CD4^+^ T cell supernatants derived from WT CD4^+^ T cells (positive control), Il3^KO^ CD4^+^ T cells treated with α-IL-5 and α-GM-CSF antibodies (test), and TSLPR^KO^ CD4^+^ T cells (negative control) also supplemented with 5 μg/ml insulin (Sigma-Aldrich). All T cells were stimulated with one round of α-CD3, α-CD28, and TSLP.

### Protein array

CD4^+^ T cell culture supernatants were thawed, and total protein concentrations were measured with a Pierce BCA Protein Assay Kit (Thermo Fisher Scientific) according to manufacturer’s instructions. Optical densities were measured on a Synergy Neo2 (BioTek) at 562 nm. Measurements were normalized, and total protein concentrations were calculated with a four-parameter logistic curve using Gen5 Microplate Reader and Imager Software (BioTek). Equal amounts of total protein in CD4^+^ T cell culture supernatants were used to screen and quantify cytokine production using the Proteome Profiler Mouse XL Cytokine Array (R&D Systems) following manufacturer instructions. Spot intensity was quantified using the Dot-Blot-Analyzer macro (written by Gilles Carpentier, 2008; available at http://rsb.info.nih.gov/ij/macros/toolsets/Dot%20Blot%20Analyzer.txt, more information can be found at http://image.bio.methods.free.fr/dotblot.html) written for ImageJ (v2.0.0).

### Western blot

Mouse breast tumor tissues were processed for protein extraction as previously described. An equal amount of protein from each sample was used for SDS-PAGE and Western blotting. After transferring to PVDF membranes, the samples were subjected to immune-blot with anti–E-cadherin, anti-Vimentin, anti-MUC1, anti-p21, anti–β-casein, anti-p53, and anti-GAPDH antibodies (Table S1) in 3% BSA in PBS. Quantification of the Western blot bands was performed using ImageJ software. Background intensity was subtracted from band intensity to determine final protein quantity for each band. GAPDH levels were used to normalize total protein levels.

### Genotyping

PCR was used to genotype genetically engineered mice. Primer pairs used in this study are described in Table S2. All primers shown are 5′ to 3′.

### RNA sequencing

Mouse CD4^+^ T cell and mouse breast tumor total RNA samples were sent to Beijing Genomics Institute for RNA sequencing. Libraries were prepared by Beijing Genomics Institute, quantified and qualified using the Agilent 2100 Bioanaylzer and ABI StepOnePlus Real-Time PCR System, and sequenced using Illumina HiSeqTM 2000. RNA sequencing data were analyzed using Pipeline v5.0. Sequences were aligned to the mouse reference genome (mm10) using Bowtie, and differentially expressed genes were screened using the Poisson distribution method, Noiseq, or EBSeq packages. Original data are available at the National Center for Biotechnology Information Gene Expression Omnibus, accession nos. GSE147067 and GSE147106.

### Gene set enrichment analysis

Gene set enrichment analysis was performed using GSEA software (Mootha et al., 2003). RNA-sequencing data were analyzed for gene set enrichment in Tslp-PyMt^tg^ Rag1^KO^ + CD4^+^ T cells and Tslp-PyMt^tg^ (test group) compared with PyMt^tg^ Rag1^KO^ + CD4^+^ T cells and PyMt^tg^ breast tumors (control group).

### ChIP-seq

ChIP assays was performed on tumor samples from PyMt Rag1^KO^ + CD4^+^ T cell and Tslp-PyMt^tg^ Rag1^KO^ + CD4^+^ T cell mice as previously described (Boulay et al., 2017; Ku et al., 2008; Mikkelsen et al., 2007). 10–30 mg of tumor was cut on dry ice and minced on ice with a razor blade. Samples were then resuspended in 1 ml cold PBS and fixed in 1% formaldehyde for 15 min at room temperature. Glycine was added for 5 min at room temperature. Samples were washed and resuspended in cold PBS and homogenized with a syringe. Chromatin extracted from formalin-fixed cells was fragmented with a Branson 250 sonifier, solubilized, and immunoprecipitated with H3K27ac antibody (Active Motif) overnight at 4°C. Antibody-chromatin complexes were pulled down with protein G-Dynabeads (Life Technologies), washed, and eluted. Immunoprecipitated DNA was extracted with AMP Pure beads (Beckman Coulter) after crosslink reversal, RNase A, and proteinase K treatment. ChIP DNA was quantified with Qubit. Sequencing libraries were prepared using 2–5 ng ChIP DNA and sequenced using the Nextseq 500 Illumina genome analyzer. ChIP-seq sequences were aligned to the mouse reference genome (mm10) using BWA (Li and Durbin, 2009). Aligned reads were extended to 200 bp to approximate fragment sizes, and density maps were created by counting the number of overlapping fragments with the Integrative Genomics Viewer (IGV) density tools (Robinson et al., 2011). ChIP-seq coverage was visualized with IGV (Thorvaldsdottir et al., 2013). Peaks were identified with the MACS2 peak caller (Zhang et al., 2008) using matched input controls and q values of 10^−2^. Pathway enrichment analysis was performed using gene set enrichment analysis computational method (http://www.gsea-msigdb.org). Peaks of acetylation in target genes were visualized using IGV (http://software.broadinstitute.org/software/igv/). Original data are available at National Center for Biotechnology Information Gene Expression Omnibus, accession no. GSE156117.

### RNA in situ hybridization

RNA in situ hybridization was performed using RNAscope probes and following manufacturer instructions (Wang et al., 2012), on formalin-fixed, paraffin-embedded tissues. The HybEZ hybridization system was used to perform RNAscope assay hybridization and incubation steps. In detail, 5-μm sections were incubated at 60°C for 1 h, deparaffinized in xylene, and rehydrated in ethanol series. Slides were placed in RNAscope 1× Target Retrieval Reagent (Advanced Cell Diagnostics) at 102°C for 15 min and treated with RNAscope Protease Plus (Advanced Cell Diagnostics) for 30 min at 40°C in a HybEZ Oven II (Advanced Cell Diagnostics). After hybridization with TSLP probe (cat no. 403541), preamplifier and amplifier, sections were stained with Fast RED reagent (RNAscope 2.5 HD Detection Reagents—RED; Advanced Cell Diagnostics), and counterstained in 50% hematoxylin and 0.02% ammonia water. RNA in situ hybridization signal was evaluated under a bright-field microscope.

### Human Protein Atlas

Immunohistochemistry staining of human IL-3Rα images are courtesy of Human Protein Atlas (Uhlen et al., 2015). Images were downloaded from the Human Protein Atlas website (https://v18.proteinatlas.org). Images can be found at the following links: https://v18.proteinatlas.org/ENSG00000185291-IL3RA/tissue/breast#img and https://v18.proteinatlas.org/ENSG00000185291-IL3RA/pathology/breast+cancer#img.

### TCGA/GTEx RNA expression data

RNA-sequencing expression data from breast cancer and normal samples were obtained from the public database Gene Expression Profiling Interactive Analysis database (GEPIA2) at http://gepia2.cancer-pku.cn/#index (Tang et al., 2017). High (top 30%) and low (bottom 30%) *TSLP*-expressing human breast cancers from TCGA dataset were compared for overall and disease-free survival. Expression of *TSLP*, *CSF2*/*IL3*/*IL5*, and differentiation genes *CSN2* (β-casein), *PRL* (prolactin), *LTF* (lactotransferrin), *LALBA* (α-lactalbumin), and *KLK10* (kalikrein-10) were examined in human breast cancers represented in TCGA.

### Statistical analysis

Graphs and statistical analysis were performed using GraphPad Prism 9 and RStudio. Bar graphs show mean + SD. Log-rank test was used to compare group survival and time to tumor onset. Two-tailed Fisher’s exact test was used to compare tumor grade distribution among groups. The nonparametric paired Wilcoxon test was used for TSLP levels in human cancer and normal tissue. One-way ANOVA was used for human TSLP RNA expression analysis, and Spearman’s rank correlation was used for gene expression correlation analyses from TCGA/GTEx datasets (GEPIA 2; Tang et al., 2019). Two-way ANOVA with Sidak’s multiple comparison test was used to compare tumor growth over time between different groups. A two-tailed unpaired *t* test was used to compare groups when the sample size was >30; two-tailed Mann–Whitney *U* test was used for all the other comparisons. A P value of <0.05 was considered significant. All error bars represent SD.

### Online supplemental material

Fig. S1 relates to Fig. 1, showing the characteristics of TSLP-induced CD4^+^ T cell immunity against spontaneous breast carcinogenesis. Fig. S2 relates to Figs. 2 and 3, showing the suppression of EMT and induction of breast cancer differentiation by TSLP-activated CD4^+^ T cell immunity. Fig. S3 relates to Figs. 4, 5, and 6, showing the critical roles of Th2 polarization and cytokine release in TSLP-stimulated CD4^+^ T cell immunity against breast cancer. Fig. S4 relates to Fig. 6, showing IL-3, IL-5, and GM-CSF receptor expression and function in breast normal epithelial and cancer cells. Fig. S5 relates to Figs. 7, 8, and 9, showing the impact of topical TSLP induction and Th2 cell immunity against mouse and human breast cancer. Table S1 lists antibodies used in the study. Table S2 lists primers used for mouse genotyping.

## Supplementary Material

Table S1 lists antibodies used in the study.

Table S2 lists primers used for mouse genotyping.
