# Supplementary material for: CD4+ T helper 2 cells suppress breast cancer by inducing terminal differentiation
Source: J Exp Med. 2022 Jun 3;219(7):e20201963. doi: 10.1084/jem.20201963 (PMC9170526; doi:10.1084/jem.20201963)
Supplement: Table S1 — lists antibodies used in the study. [file JEM_20201963_TableS1.docx]

**Table S1. Antibodies used in the study**

| **Flow cytometry Antibodies** | **Conjugate** | **Clone** | **Manufacturer** | **Cat #** | **Isotype** | **RRID** |
| --- | --- | --- | --- | --- | --- | --- |
| CD3 | Alexa Fluor 700 | 17A2 | BioLegend | 100216 | Rat IgG2b, κ | AB_493697 |
| CD3 | FITC | 145-2C11 | eBioscience | 11-0031-85 | Armenian Hamster IgG | AB_464883 |
| CD4 | APC-Cy7 | RM4-5 | BioLegend | 100526 | Rat IgG2a, κ | AB_312727 |
| CD8a | PE-Cy7 | 53-6.7 | BioLegend | 100722 | Rat IgG2a, κ | AB_312761 |
| CD8a | FITC | 53-6.7 | Biolegend | 100706 | Rat IgG2a, κ | AB_312745 |
| CD19 | PE | 1D3/CD19 | BioLegend | 152408 | Rat IgG2a, κ | AB_2629817 |
| CD49b | PE | DX5 | BioLegend | 108907 | Rat IgM, κ | AB_313414 |
| NK1.1 | PE | PK136 | BioLegend | 108708 | Mouse IgG2a, κ | AB_313395 |
| NKp46 | PerCP-Cy5.5 | 29A1.4 | BioLegend | 137609 | Rat IgG2a, κ | AB_10642684 |
| NKp46 | FITC | 29A1.4 | BioLegend | 137606 | Rat IgG2a, κ | AB_2298210 |
| CD19 | eFluor 450 | eBio1D3 | eBioscience | 48-0193-82 | Rat IgG2a, κ | AB_2734905 |
| CD45.1 | APC | A20 | eBioscience | 17-0453-82 | Mouse IgG2a, κ | AB_469398 |
| GATA3 | PerCP-Cyanine5.5 | 16E10A23 | BioLegend | 653812 | Mouse IgG2b, κ | AB_2563219 |
| Foxp3 | APC | FJK-16s | eBioscience | 17-5773-82 | Rat IgG2a, κ | AB_469457 |
| Ki67 | eFluor 450 | SolA15 | eBioscience | 48-5698-82 | Rat IgG2a, κ | AB_11149124 |
| Ki67 | Brilliant Violet 605 | 16A8 | BioLegend | 652413 | Rat IgG2a, κ | AB_2562664 |
| CD123 | PE | 5B11 | BioLegend | 106005 | Rat IgG2a, κ | AB_2124403 |
| CD131 | PE | JORO50 | BD Biosciences | 559920 | Rat IgG1, κ | AB_397374 |
| CD125 | PE/Cyanine7 | DIH37 | BioLegend | 153408 | Rat IgG2a, κ | AB_2819905 |
| CD116 | APC | 698423 | R&D Systems | FAB6130A | Rat IgG2a | AB_10973836 |
| EpCAM | APC | G8.8 | BioLegend | 118213 | Rat IgG2a, κ | AB_1134105 |
| CD45 | Brilliant Violet 421 | 30-F11 | BioLegend | 103133 | Rat IgG2b, κ | AB_10899570 |
| **Immunofluorescence and Immunohistochemistry Antibodies** | **Conjugate** | **Clone** | **Manufacturer** | **Cat #** | **Isotype** | **RRID** |
| CD3 | Purified | CD3-12 | Abcam | Ab11089 | Rat IgG1 | AB_369097 |
| CD4 | Purified | EPR19514 | Abcam | Ab183685 | Rabbit IgG | AB_2686917 |
| CD8 | Purified | D4W2Z | Cell Signaling Technologies | 98941 | Rabbit IgG | AB_2756376 |
| E-cadherin | Purified | 36/E-Cadherin | BD Biosciences | 610181 | Mouse IgG2a, κ | AB_397580 |
| Ki67 | Purified | Polyclonal | Abcam | Ab15580 | Rabbit IgG | AB_443209 |
| Cleaved Caspase 3 | Purified | 5A1E | Cell Signaling Technologies | 9664 | Rabbit IgG | AB_2070042 |
| CD103 | Purified | EPR22590-27 | Abcam | 224202 | Rabbit IgG |  |
| MHCII | Purified | M5/114.15.2 | Thermo Fisher Scientific | 16-5321-85 | Rat IgG2b, κ | AB_469099 |
| MCP-8 | Purified | TUG8 | BioLegend | 647401 | Rat IgG2a | AB_2069309 |
| CD11c | Purified | DIV9Y | Cell Signaling Technologies | 97585 | Rabbit IgG | AB_2800282 |
| TSLP | Purified | 73AD11 | Merck | GNE01.12F3.B5.4011 | Rat IgG2a |  |
| β chain (IL-3RB) C-terminal | Purified | Polyclonal | Abcam | Ab198701 | Rabbit IgG |  |
| GATA3 | Purified | HG3-31 | Santa Cruz | sc-268 | Mouse IgG1 | AB_2108591 |
| Cytokeratin | Purified | AE1/AE3 | Dako | M3515 | Mouse IgG1 | AB_2132885 |
| Ovalbumin | Purified | Polyclonal | Abccam | Ab181688 | Rabbit IgG |  |
| **Blocking Antibodies** | **Conjugate** | **Clone** | **Manufacturer** | **Cat #** | **Isotype** | **RRID** |
| *InVivo*MAb anti-mouse GM-CSF | Purified | MP1-22E9 | BioXCell | BE0259 | Rat IgG2a, κ | AB_2687738 |
| *InVivo*MAb anti-mouse/human IL-5 | Purified | TRFK5 | BioXCell | BE0198 | Rat IgG1, κ | AB_10950522 |
| **T cell stimulation Antibodies** | **Conjugate** | **Clone** | **Manufacturer** | **Cat #** | **Isotype** | **RRID** |
| InVivo*MAb anti-mouse CD3ε* | Purified | 145-2C11 | BioXCell | BE0001-1 | Armenian Hamster IgG1 | AB_1107634 |
| InVivo*MAb anti-mouse CD28* | Purified | 37.51 | BioXCell | BE0015-1 | Syrian Hamster IgG2 | AB_1107624 |
| **Western blotting** | **Conjugate** | **Clone** | **Manufacturer** | **Cat #** | **Isotype** | **RRID** |
| E-cadherin | Purified | 36/E-Cadherin | BD Biosciences | 610181 | Mouse IgG2a, κ | AB_397580 |
| Vimentin | Purified | D21H3 | Cell Signaling Technologies | 5741 | Rabbit IgG | AB_10695459 |
| MUC1 | Purified | EP1024Y | Abcam | ab45167 | Rabbit IgG | AB_776552 |
| p21 | Purified | F-5 | Santa Cruz Biotechnology | sc-6246 | Mouse IgG2b, κ | AB_628073 |
| Beta-casein | Purified | H-4 | Santa Cruz Biotechnology | sc-166530 | Mouse IgG2a, κ | AB_2084348 |
| P53 | Purified | FL-393 | Santa Cruz Biotechnology | sc-6243 | Rabbit IgG | AB_653753 |
| GAPDH | Purified | D16H11 | Cell Signaling Technologies | 5174 | Rabbit IgG | AB_10622025 |
