# Supplementary material for: CD4+ T helper 2 cells suppress breast cancer by inducing terminal differentiation
Source: J Exp Med. 2022 Jun 3;219(7):e20201963. doi: 10.1084/jem.20201963 (PMC9170526; doi:10.1084/jem.20201963)
Supplement: Table S2 — lists primers used for mouse genotyping. [file JEM_20201963_TableS2.docx]

**Table S2. Primers used for mouse genotyping**

| **Gene** | **Forward primer (5′–3′)** | **Reverse primer (5′–3′)** |
| --- | --- | --- |
| Tslp^tg^ | TCATCCTGCAAGTACTAGTACGGATGGGGC | TGTTTTGGACTTCTTGTGCCATTTCCTGAG |
| PyMt^tg^ | ATACTGCTGGAAGAAGACGAAATCCTTG | CTCTGTGAGTAGCTCTCATTCTCTGACTC |
| Brca^wt^ | GGTGCTCTCAAGGGCCATGATTGTCAGTTC | ATGGGTGAGCGATGGAAGCTCCTTCACCAC |
| Brca^flox^ | GGTGCTCTCAAGGGCCATGATTGTCAGTTC | GTCAGCAAAGCCCGGGTTTGGT |
| K14^Cre^ | GCATTACCGGTCGATGCAACGAGTGATGAG | GAGTGAACGAACCTGGTCGAAATCAGTGCG |
| P53^flox^ | AAACAGGTTAAACCCAGCTTGACCAAGTGC | ACATAGGAGGCAGAGACAGTTGGAGGCCAG |
| Il4r^KO^ | GGTTGCAGGGAACAGCCCAGAAAAGTGAAG | CCAGACTGCCTTGGGAAAAG |
| Il4r^wt^ | GGTTGCAGGGAACAGCCCAGAAAAGTGAAG | TCCTCTGTGGGCTCAGAGTGACCATGAGAA |
| Her2 | CCCCGGGAGTATGTGAGTGA | TGAGCTGTTTTGAGGCTGACA |
| Tslpr^KO^ | AGCGTTGGCTACCCGTGATATTGCTGAAGAG | TCATGAACGACCACTTCCTATGTTGGACACG |
| Rag1^wt^ | CCAGTAGATACCATTGCGAAGAGG | CACGTTCTGTGAACCATGCTCTATC |
| Il3^wt^ | GGGTTTTTGGCATCTTGGT | CCAGGGAGATGAGATCCAGA |
| Il3^KO^ | AAGGGGCCACCAAAGAAC | GGGTTTTTGGCATCTTGGT |
| Il4^wt^ | GTGAGCAGATGACATGGGGCAGAAAAAACC | TCTTCTTCAAGCATGGAGTTTTCCCATGTT |
| Il4^KO^ | GTGAGCAGATGACATGGGGCAGAAAAAACC | GCCGATTGTCTGTTGTGCCCAG |
| IFNg^wt^ | AGAAGTAAGTGGAAGGGCCCAGAAG | AGGGAAACTGGGAGAGGAGAAATAT |
| IFNg^KO^ | CCTTCTATCGCCTTCTTGACG | AGGGAAACTGGGAGAGGAGAAATAT |
| TNFa^wt^ | GGGACTAGCCAGGAGGGAGAACAGAAACTC | GGGGGAGTGCCTCTTCTGCCAGTTCCACGT |
| TNFa^KO^ | GGGACTAGCCAGGAGGGAGAACAGAAACTC | CGTTGGCTACCCGTGATATT |
| TCRa | CAATAAAAGGGAGAAAAAGCTCTCCTTGCA | CTGGTACACAGCAGGTTCTGGGTTCTGGAT |
| TCRb | CTGCTGCTGCTGCACAGACCTACTCTTTC | CAGCTCACCTAACACGAGGAGCCGAGTGCC |
